# Formulation and *In Vitro*, *In Vivo* Evaluation of Effervescent Floating Sustained-Release Imatinib Mesylate Tablet

**DOI:** 10.1371/journal.pone.0126874

**Published:** 2015-06-02

**Authors:** Ali Kadivar, Behnam Kamalidehghan, Hamid Akbari Javar, Ehsan Taghizadeh Davoudi, Nurul Dhania Zaharuddin, Bahareh Sabeti, Lip Yong Chung, Mohamed Ibrahim Noordin

**Affiliations:** 1 Department of Pharmacy, Medical Faculty, University of Malaya, Kuala Lumpur, Malaysia; 2 Department of Pharmaceutics, Faculty of Pharmacy, Tehran University of Medical Sciences (TUMS), Tehran, Iran; Taipei Medical University, TAIWAN

## Abstract

**Introduction:**

Imatinib mesylate is an antineoplastic agent which has high absorption in the upper part of the gastrointestinal tract (GIT). Conventional imatinib mesylate (Gleevec) tablets produce rapid and relatively high peak blood levels and requires frequent administration to keep the plasma drug level at an effective range. This might cause side effects, reduced effectiveness and poor therapeutic management. Therefore, floating sustained-release Imatinib tablets were developed to allow the tablets to be released in the upper part of the GIT and overcome the inadequacy of conventional tablets.

**Methodology:**

Floating sustained-release Imatinib mesylate tablets were prepared using the wet granulation method. Tablets were formulated using Hydroxypropyl Methylcellulose (HPMC K4M), with Sodium alginate (SA) and Carbomer 934P (CP) as release-retarding polymers, sodium bicarbonate (NaHCO_3_) as the effervescent agent and lactose as a filler. Floating behavior, *in vitro* drug release, and swelling index studies were conducted. Initial and total drug release duration was compared with a commercial tablet (Gleevec) in 0.1 N HCl (pH 1.2) at 37 ± 0.5°C for 24 hours. Tablets were then evaluated for various physical parameters, including weight variation, thickness, hardness, friability, and drug content. Consequently, 6 months of physical stability studies and *in vitro* gastro-retentive studies were conducted.

**Results and Discussion:**

Statistical data analysis revealed that tablets containing a composition of 14.67% w/w HPMC K4M, 10.67%, w/w Na alginate, 1.33%, w/w Carbomer 934P and 9.33%, w/w NaHCO_3_ produced the most favorable formulation to develop 24-hour sustained-release tablets with optimum floating behavior and satisfactory physicochemical characteristics. Furthermore, *in vitro* release study revealed that the formulated SR tablet had significantly lower C_max_ and higher T_max_ compared to the conventional tablet (Gleevec). Thus, formulated SR tablets preserved persistent concentration of plasma up to 24 hours.

**Conclusion:**

In conclusion, in order to suggest a better drug delivery system with constant favorable release, resulting in optimized absorption and less side effects, formulated CP-HPMC-SA based imatinib mesylate floating sustained-release tablets can be a promising candidate for cancer chemotherapy.

## Introduction

The main challenge in developing an oral controlled-release drug delivery system is sustaining the drug release and maintaining the dosage form in the gastrointestinal tract (GIT) for an extended period of time [1]. The major limitation of most currently-available oral drug delivery systems is a fast gastric-emptying time. Therefore, the past few decades have seen an increased interest in gastric drug retention [2]. Gastroretentive dosage forms are classified into four main classes: bioadhesive system [3], floating system [4], high-density system [5] and expandable system [6]. Imatinib is known to be slightly soluble to insoluble in neutral and alkaline aqueous conditions [7]. Therefore the floating system, which prolong the gastric retention of the tablet to release the drug in a sustained form in the acidic part of the GIT, is produced. In this study, the tablets will be developed using the effervescent system, whereby carbon dioxide gas is generated upon contact of tablets with gastric fluid, which would influence the tablets to float [8]. The Sustained-release (SR) oral drug delivery system is proven to be effective in achieving optimal drug plasma concentration through the consumption of a single dose while maintaining the therapeutic value in the blood throughout its desired period of time [9].

**Fig 1:**
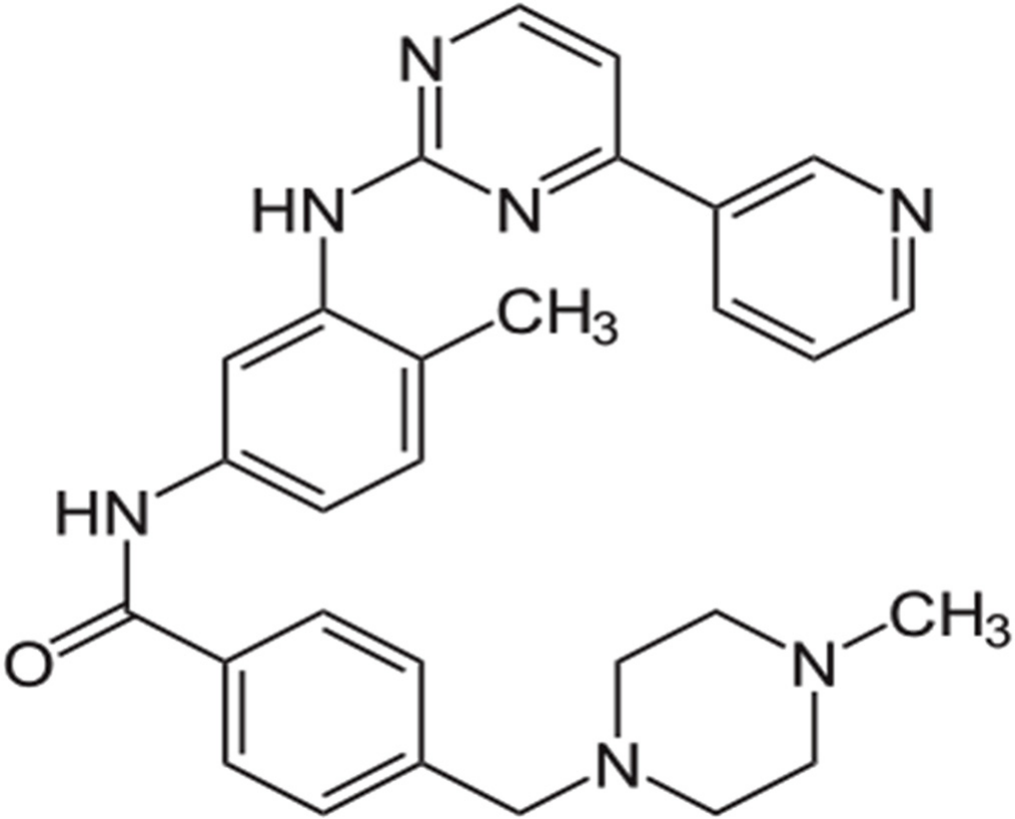
The chemical structure of Imatinib mesylate.

Imatinib is a small molecule with antineoplastic effects (Fig 1). It functions as a specific inhibitor for a number of tyrosine kinase enzymes and acts by binding to the site of tyrosine kinase while preventing its activity, leading to apoptosis [10]. Imatinib mesylate (Gleevec, also known as STI–571), has been authorized to treat chronic myelogenous leukemia [11], gastrointestinal stromal tumors [12] and is an important therapeutic period of breast cancer as well as some other types of cancer [13]. Its adverse effects include hair loss, diarrhea, nausea, loss of appetite, vomiting, dry skin, muscle cramps and swelling (especially in the legs or around the eyes) [14]. Therefore, sustained release tablets were developed to prevent these side effects while floating tablets release the drug locally in the absorbent part in a sustained form. [15].

Imatinib is a 2-phenylaminopyrimidine derivative with a pKa value of 12.45 in acidic environment and 8.27 in basic environment [16]. It is soluble in conditions of less than pH 5.5 but is very slightly soluble to insoluble in neutral and alkaline aqueous buffers [17]. It has a high absorption in the upper part of the gastrointestinal tract (GIT), thus a floating sustained-release tablet of imatinib mesylate was formulated to be retained in the stomach and achieve appropriate release and absorption. The elimination half-lives after administration are approximately 13–16 hours for Imatinib [18], and 40 hours for its major active metabolites, the N-desmethyl derivative. [16, 17]

Anjali Dev et. al [19], conducted a short study to formulate 12-hour floating controlled-release tablet of 120 mg imatinib mesylate using HydroxyPropyl MethylCellulose (HPMC) and Sodium BiCarbonate (SBC); but oral imatinib mesylate is administered at dosages of 400, 600 or 800 mg daily [20, 21]. Therefore, 24-hour floating sustained-release tablet of 400 mg imatinib mesylate was formulated in this study to provide for all the orally-administered dosages of imatinib mesylate, making it a once-daily (24 h) dosage to improve drug efficiency and patient compliance. Using only HPMC to formulate the floating tablet, the tablet disintegrated after few hours (less than 3hours); therefore a 24-hour effervescent floating sustained-release tablet of 400 mg imatinib mesylate was formulated and the *in vivo* gastroretentive properties of the formulated tablet were investigated.

The objective of the present investigation was to design a promising imatinib mesylate effervescent floating sustained-release tablet by utilizing release-retarding gel-forming polymers such as hydroxypropyl methylcellulose (HPMC K4M), sodium alginate (Na alginate), carbomer, as well as an effervescent agent, sodium bicarbonate (NaHCO_3_). Optimal formulation was designed to attain consistent 24-hour controlled-release tablets with good floating behavior and favorable physical tablet characteristics.

## Materials and Methods

### Materials

Imatinib mesylate was kindly provided by Osvah Pharmaceutical Company (Tehran, Iran). HPMC K4M and microcrystalline cellulose (MCC) (Avicel pH 101) were supplied by Sigma-Aldrich (M) Sdn Bhd (Subang jaya, Malayisa). Low viscosity sodium alginate and sodium bicarbonate were purchased from R&M chemicals (KL, Malaysia). Carbomer 934P was obtained from Noveon (USA), polyethylene glycol 3500 from Merck (Petaling Jaya, Malaysia), magnesium stearate from Mallinckrodt (Dublin, Ireland) and lactose monohydrate from HMbG chemicals (Germany). All other reagents are of analytical or pharmaceutical grade and deionized water was obtained by reverse osmosis.

### Preparation of imatinib mesylate floating sustained-release tablets (FSRT)

Tablets containing 400 mg of imatinib mesylate were prepared by the wet granulation method using isopropyl alcohol as a granulating agent [22]. The ingredients were accurately weighed and mixed thoroughly according to Table 1. Eight individual formulations were then passed through a “No. 20” mesh sieve, and uniformly wetted by spraying 5% w/w isopropyl alcohol. The wet mass was mixed consistently, passed through a “No. 20” mesh screen and dried at 50°C in a hot dry oven for 30 min. After drying, the granules were then further sized by passing through a “No. 30” sieve and mixed with 10 mg of PEG 3500 and 5 mg of magnesium stearate as a lubricant to promote easy ejection and good anti-sticking property [23].

**Table 1:** The composition (mg) of the investigated Imatinib mesylate FSR tablets.

Formula code	Drug	HPMC K4M	Na alginate	Carbomer 934P	Na bicarbonate	PEG 3500	Mg stearate	Lactose
**F1**	400	150	50	20	70	10	5	45
**F2**	400	130	70	20	70	10	5	45
**F3**	400	110	90	20	70	10	5	45
**F4**	400	90	110	20	70	10	5	45
**F5**	400	110	90	10	70	10	5	55
**F6**	400	110	80	10	70	10	5	65
**F7**	400	110	70	10	70	10	5	75
**F8**	400	110	60	10	70	10	5	85

Finally, 750 mg of each formulation was weighed and fed manually into the die of a single punch tableting machine (Globepharma MTCM-1, ENER PAC), using 12.0mm flat-faced punches with a compression force of 250 kg/cm^2^ [24]. At least thirty tablets were prepared for each formulation in a single batch.

### Physical characterization and *in vitro* evaluation of the prepared tablets

Physical characterization and *in vitro* study of the prepared tablets was conducted using British Pharmacopeia (BP 2010) and USP standard procedures [25, 26].

#### Weight variation study

Twenty tablets were randomly selected and then an electronic balance (Mettler Toledo, 3-MS-S/MS-L, Switzerland) was used to accurately weigh them. Results were reported as mean values ± SD (Standard Deviation) [27].

#### Thickness study

Ten tablets were randomly selected and the thickness was measured using a Vernier caliper. Results were expressed as mean values ± SD [28].

#### Hardness study

The tablet’s hardness (n = 5) was investigated using Dr. Schleuniger, 6D Tablet Tester (Pharmatron, Switzerland). The hardness of tablets was reported as mean values ± SD [29].

#### Drug content uniformity

Ten tablets were individually weighed and crushed. Each tablet’s powder (750 mg) was dissolved in 100 mL of 0.1N HCl. The solution was filtered through a cellulose acetate membrane (0.45 μm) and the drug content was determined by UV spectroscopy (Perkin Elmer, Lambda 25, USA) at a wavelength of 237 nm after suitable dilution with 0.1 N of HCl [30].

#### Friability study

Ten tablets were randomly subjected to the tablet friability test. According to the BP specifications [31], tablets were loaded in the tablet friability test apparatus drum (ERWEKA, TAR 10, Germany) and rotated for 100 cycles for 4 minutes. The tablets were removed, dedusted and accurately weighed. The percent weight loss was calculated [30].

#### Tablet swelling behavior

The method by Dorozynski et al. was used to determine the tablets’ swelling behavior in this study, which was done in triplicates [32]. A weighed tablet (W1) was placed in a glass beaker which contained 900 mL of 0.1N HCl, and was kept at 37 ± 0.5°C in a water bath. The swollen tablet was taken out of the solution at determined time intervals, and after being dried of excess liquid by dabbing with a filter paper, was reweighed (W2) [33]. Eq 1 was used to calculate the swelling index (S1).

SI=(W2−W1)/W1 (1)

#### Tablet floating behavior

The floating study was conducted using the USP dissolution apparatus II, where each tablet was placed in a beaker containing 900 mL of 0.1N HCl, which was kept at 37 ± 0.5°C in a water bath. The floating lag-time, which is described as the time taken for the tablet to float, and the total floating time (the duration which the tablet remains buoyant) were recorded [34–36].

#### Tablet Physical stability study

The International Conference on Harmonization (ICH) guidelines were used as a reference for the physical stability studies [37]. F6, which was the best formula, was stored in tightly-capped polyethylene bottles and kept in a desiccator which contained saturated sodium chloride solution (75% RH) [38]. The desiccator was placed in oven at 40°C for six months. At pre-determined time intervals, the stored tablet was studied for the following parameters: drug content, hardness value and floating behavior and matrix integrity, which was observed visually [39]. Statistical analysis was done using paired Student’s t-test, at a significance of p<0.05.

### *In vitro* dissolution study

The *in vitro* release rate of imatinib mesylate from Imatinib FSR tablets (n = 3) was determined using a dissolution machine (ERWEKA DT 70, Germany). The dissolution test was performed using 900 mL of 0.1N HCl (pH 1.2) at 37 ± 0.50°C and 75 rpm according to the United States Pharmacopeia (USP) Dissolution testing apparatus II (paddle) method [26]. A sample (10 mL) of the solution was withdrawn from the dissolution apparatus hourly for the first 12 hours and every 2 hours for the next 12 hours. The withdrawn volume was replaced with 10ml of fresh dissolution medium. A 0.45μm membrane filter was used to filter the samples, which were then diluted with 0.1N HCl to an appropriate concentration. The solutions’ absorbance was then measured using a UV spectrophotometer (Perkin Elmer, Lambda 25, USA) at 237 nm. Cumulative percentage drug release was calculated based on the standard curve [40]. The 10-concentration standard curve between 50–600 μg/ml was linear (r2 > 0.998), with the mean of 2.19% inter- and intra-day precision and accuracy, 5 μg/ml detection limit and 97.4% average for 5 separate serial dilution recoveries applied for the calibration of standard concentrations.

To study the drug release for Gleevec the same procedure was applied, with successive 5-minute interval sampling for up to 30 minutes [40].

#### Kinetic modeling of drug release profiles

The dissolution profiles of all formulae in 0.1 N HCl were studied by plotting the data obtained from their *in vitro* drug release to Zero-order, First-order, Higuchi Hixson–Crowell, Korsmeyer-Peppas [41] and Weibull kinetic models [42]. The model with the highest correlation coefficient (R^2^) was considered to be the best fit for the designated kinetic release [43].

Zero-order (Eq 2) data is plotted as cumulative percentage of drug released against time.

$${\text{C}}_{\text{t}}={\text{C}}_{0}+{\text{K}}_{0}\text{t}$$ (2)

Where C represents the concentration, K_0_ is the zero-order rate constant expressed as concentration/time, and t is time in hours.

First-order (Eq 3) data was plotted as the log cumulative percentage of released drug against time.

$${\text{log C}}_{\text{t}}={\text{log C}}_{0}-\left({\text{k}}_{1}\text{t}\;/\;2.303\right)$$ (3)

Where C_0_ represents the initial drug concentration, k_1_ is the first order rate constant, and t is the time in hours.

Data was plotted as the cumulative percentage released drug against the square-root of time according to Higuchi’s equation (Eq 4)

$${\text{C}}_{\text{t}}={\text{K}}_{\text{H}}{\text{t}}^{1/2}$$ (4)

Where K_H_ represents the constant of the system, and t is the time.

Moreover, for better characterization of the drug release mechanisms, the Hixson-Crowell model (Eq 5) was applied:

$${\text{C}}_{0}{}^{1/3}-{\text{C}}_{\text{t}}{}^{1/3}={\text{K}}_{\text{H}}\text{t}$$ (5)

Where C_0_ is the initial amount of drug in the pharmaceutical dosage form, C_t_ is the remaining amount of drug in the pharmaceutical dosage form at time t and K_H_ stands for the Hixson-Crowell rate constant.

The Weibull equation is commonly used in dissolution studies and can be applied for all types of dissolution curves (Eq 6). The accumulated fraction of drug release, m, in solution at time t, is expressed as:

$$\text{m}=1-\exp[-{\left(\text{t}-{\text{T}}_{\text{i}}\right)}^{\text{b}}(1/\text{a})]$$ (6)

where a is the time process, T_i_ is the lag time, in most cases zero, and b, the shape parameter, characterizes the curve as exponential (b = 1), S-shaped with upward curve followed by the turning point (b > 1), or parabolic with a higher initial slope, which after that is consistent with the exponential curve (b < 1).

The rearranged form of Eq 6 is:

$$\text{log}\;\left[-\text{ln}\;\left(1-\text{m}\right)\right]=\text{b log}\;\left(\text{t}-{\text{T}}_{\text{i}}\right)-\text{log a}$$ (7)

Drug release following this model will be linear when the log dissolved amount of drug plot vs. the log of time (Eq 7).

### Drug-release profile statistical analysis

One-way ANOVA using SPSS 20.0.0 (SPSS Inc., Chicago, USA) software was applied to analyze the data. Post-hoc multiple comparisons using least-square difference (LSD) was carried out with a p < 0.05 statistical significance.

### *In vivo* gastro-retentive X-ray study

Five males and four females, nine healthy, ~15 weeks old, 2–2.5 kg, New Zealand Albino rabbits were used in this study. The rabbits were kept in separate cages in room temperature with equal interval of light and dark i.e. 12 hours each. Fresh grass and green fodder was offered in the morning and evening, whereas fresh drinking water was provided around the clock. Rabbits were acclimatized for five days.

All animals received a thorough physical examination before entry into the study. Any animal that did not meet the health and weight criteria was excluded from the study. Animals with any inflammation, dermatitis, infection or apparent abnormalities of the urinary tract as well as any animal deemed unsuitable by the study director were also excluded from this study. At the end animals were sacrificed with 20% KCl (2 ml, i.v.). All procedures were performed in compliance with the “Guide for the Care and Use of Laboratory Animals” by the National Academy of Sciences and approved by the University Ethics Committee, University of Malaya (UM IACUC) with FAR/30/05/2012/ET ethics reference before the experiment.

To evaluate the gastro-retentive behavior of the best formulation (F6) *in vivo*, incorporation of Barium sulfate (BaSO4) was required to visualize the tablets under X-ray but not to change the tablet buoyancy, therefore 350 mg tablet was prepared with same formulation and 25% of the drug was replaced with barium sulfate with no change in other ingredients, then 7.3 mm flat-faced die used to punch tablets with a compression force of 250 kg/cm2. [44]. Then the *in vitro* tablet floating lag time and floating total time were examined and compared with the original tablet’s characterizations. After overnight fasting, six healthy rabbits were fed with a little low calorie food and given some water. One hour later, the tablet was administered orally, being placed using a “pet piller”. The tube was inserted into the rabbit’s mouth and blown using a rubber bulb, then each rabbit was fed with15 ml of water [45]. The animals were held in a standing position and at the same distance from the X-ray source to take a clear image. Radiographs were obtained at 15 minutes, and at 4, 8, 12, 18 and to a maximum of 24 h. The rabbits were given 1.5 ml of water hourly and had access to food within the 8^th^ to 9^th^ and the 18^th^ to 19^th^ hours intervals. The X-ray parameters were constant during experiment. With evaluating X-ray images the tablet positions were easily identified and observed.

### *In vivo* dissolution study

#### Animals and treatments

Twelve female rabbits (New Zealand albino) 2–2.5 kg were participate in this study. The animals were kept according to the above-mentioned conditions and code of ethic. Six rabbits were gavaged with single dosage 200 mg formulated Imatinib SR tablet (F6) and the other six with 200 mg commercial tablet (Gleevec) using the pet piller. They fasted for 12 hours before the experiment and 8 hours after the administration of the tablet, but they had free access to water. The recommended dosage (200 mg), fasting condition and blood sampling were invariants.

#### Sample preparation

For the experiment, 1–1.5 ml blood samples were collected from the ear vein into the heparinized tube at 0, 30 min, every hour within 1–4 hours and every 2 hours within 4–12 hours as well as 18, 24, 36, 48 and 72 hours after the tablet administration (fourteen time points) for both the formulated floating sustained-release and commercial tablets. To separate plasma, 4000 rpm for 10 minutes was set to centrifuge the blood samples, then the supernatant was collected and stored at −80°C. A protein precipitation process was used to analyze the plasma samples [46]. Then, 100 μL of the plasma sample was extracted and 10 mL of the sample extracts was injected into the LC-MS/MS system. To prepare for the extraction, 800 μL of ethyl acetate was added to 200 μl of plasma and vortexed for 30 seconds. Afterward, the samples were centrifuged 10 min at 14000 rpm, and the organic layer was transferred to a new tube to separate debris from the organic layer. Nitrogen steam was applied to dry the organic layer. Further, 50 μL of the dried sample was mixed with 50 μL of methanol to re-establish the sample in 100 μL of solution. Finally, samples were analyzed by LC-MS/MS [47]. The LC-MS/MS assay was validated in SUCXeS lab (University of Malaya).

#### Method development

Samples were quantitative analyzed for Imatinib mesylate and CGP74588 (its major active metabolite) through AB Sciex API 5500 LC-MS/MS systems using the Liquid Chromatography/dual Mass Spectrometry (MS/MS) assay. The MS/MS detection was done through the Electrospray-positive ionization (ESI C) multiple reaction monitoring with ion transitions of m/z 503.7 to 393.50 for the internal standard (IS), m/z 479.82 to 393.98 daughter ion for the metabolite CGP74588, and m/z 493.68 to 393.86 daughter ion for Imatinib mesylate. In this study, 0.1–100 μg/mL of linear dynamic range was found with less than 7.87% overall bias and CV. Moreover overall 0.9978 of regression coefficient (R^2^) indicated the assay linearity. Unidentified sample concentrations were computed from the fraction of the daughter ion of the analytes to its internal standard’s daughter ion (ordinate) as the peak area, against the abscissa (nominal concentration) [48].

The validation of method was done for stability, specificity, precision, linearity, recovery and accuracy consistent with the FDA principles of bioanalytical method validation guidance for industry [49].

## Results and Discussion

### Physicochemical characteristics of tablets

Controlled-release imatinib mesylate effervescent floating tablets were developed using release-retarding gel-forming polymers HPMC K4M, Na alginate and carbomer 934P, accompanied by NaHCO_3_ as a gas-forming agent and lactose as a filler. The physicochemical properties of the tablets are summarized in Table 2. The tablets showed acceptable hardness value, where previous studies have reported that tablet hardness had little or no effect on the drug release profile but may affect the buoyancy of tablets [50]. Increment of the hardness may lead to prolonged tablet floating lag-time by decelerating the rate of dissolution medium penetration of the tablet.

**Table 2:** Physicochemical properties of the prepared Imatinib mesylate FSR tablets.

Formula code	Thickness (mm)	Weight (mg)	Hardness (N)	Drug content (%)	Friability (%)
**F1**	3.98 ± 0.09	746.95 ± 2.74	95.84 ± 0.84	99.44 ± 0.86	0.12 ± 0.03
**F2**	4.07± 0.16	754.84 ± 1.25	92.65 ± 0.77	100.02 ± 0.96	0.17 ± 0.02
**F3**	3.98 ± 0.11	745.07 ± 3.02	102.72 ± 0.89	97.39 ± 0.64	0.21 ± 0.01
**F4**	4.12 ± 0.06	755.87 ± 2.17	98.66 ± 0.90	101.23 ± 0.78	0.33 ± 0.04
**F5**	4.05 ± 0.13	751.39 ± 1.9	97.53 ± 0.88	98.93 ± 1.03	0.34 ± 0.01
**F6**	3.92 ± 0.10	748.19 ± 1.46	88.68 ± 0.91	99.74 ± 0.89	0.37 ± 0.05
**F7**	4.06 ± 0.15	753.26 ± 2.28	87.56 ± 0.73	101.4 ± 1.15	0.35 ± 0.01
**F8**	4.16 ± 0.15	746.26 ± 2.28	85.06 ± 1.03	96.4 ± 1.15	0.42 ± 0.01

The thickness of all tablet batches ranged from 3.92 ± 0.10 to 4.16 ± 0.15 mm, while the weight of the tablets ranged from 745.07± 3.02 to 755.87 ± 2.17 mg with CV% less than 2%. The percentage of drug content ranged from 96.4% ± 1.15 to 101.4% ± 1.15 and the percentage friability for all formulae was less than 0.5%, indicating good mechanical resistance. All the tablet formulae showed acceptable physicochemical properties complying with USP 27 requirements [51].

### Swelling behavior

**Fig 2:**
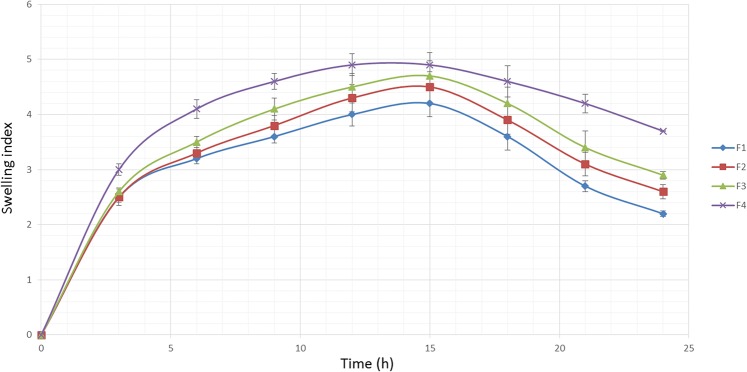
The influence of the HPMC K4M/Na alginate ratio on the swelling ability of imatinib mesylate floating tablets (mean ± SD, n = 3).

**Fig 3:**
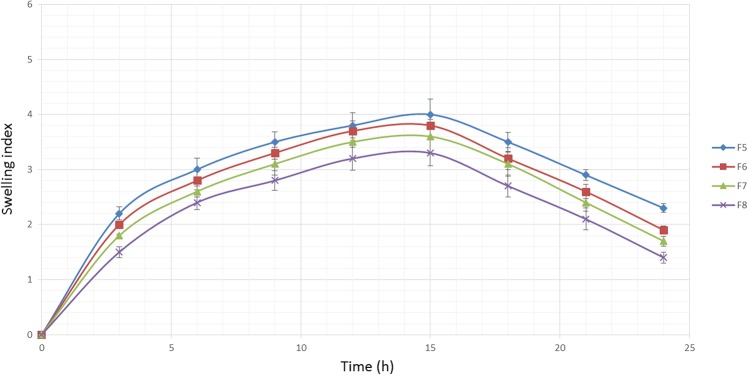
The influence of the Na alginate ratio on the swelling ability of imatinib mesylate floating tablets (mean ± SD, n = 3).

**Fig 4:**
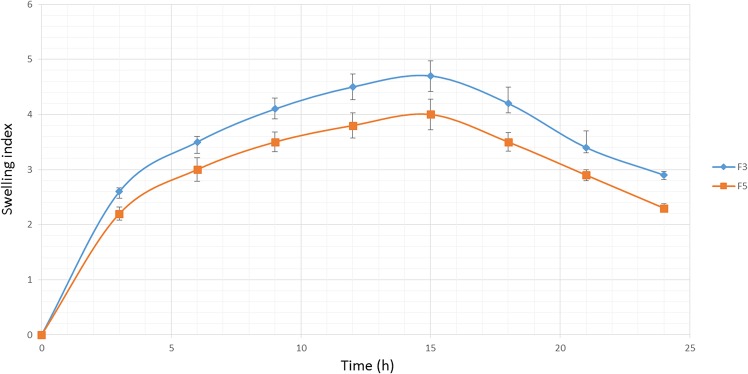
The influence of the Carbomer 934P ratio on the swelling ability of imatinib mesylate floating tablets (mean ± SD, n = 3).

The hydration ability of tablets is important as it influences tablet buoyancy, swelling behavior and drug release kinetics [52]. As can be seen from Figs 2, 3 and 4, it could be concluded that the fluid uptake of the prepared matrices depends on the type and amount of polymers in each formulation. The maximum swelling index of all formulae (5 ± 0.13) was achieved after 13 hours. As observed in Fig 2, a higher amount of HPMC K4M/Na alginate ratio, as indicated in the formula F1-F4, causes a decrease in the swelling indices. This shows that HPMC K4M does not promote swelling characteristics. An elevated swelling of tablets was observed in F5-F8 (Fig 3) upon increasing the amount of sodium alginate, where this indicates that sodium alginate contributes to the swelling of tablets. At pH 1.2, sodium alginate is rapidly converted to alginic acid, which has the ability to swell upon hydration [53]. Fig 4 shows the effect of carbomer on swelling indices, whereby a higher amount of carbomer resulted in significantly higher swelling capacity. This can be explained by the mechanism of carbomer as a matrix-forming polymer in tablets, whereby it is easily hydrated, resulting to a reduction in the glass transition temperature (Tg) to the temperature of the dissolution medium. This causes the polymer to be more volatile and is able to swell more quickly [54, 55]. A significant increase in the molecular volume of the hydrated polymer reduces the free volume of the matrix system [56], thus enabling the control and retardation of the mechanism of drug release.

Formula F4 presented the highest swelling index throughout the study period. This may be related to the influence of the high amount of Na alginate and carbomer in the formulation. On the other hand, formula F8 indicated the lowest swelling index, where it is associated with the minimum amount of carbomer and Na alginate. However, high swelling indices do not necessarily control and retard the release of the drug, as can be seen from Fig 3, where F4, with the highest amount of Na alginate, had the highest drug release rate.

**Fig 5:**
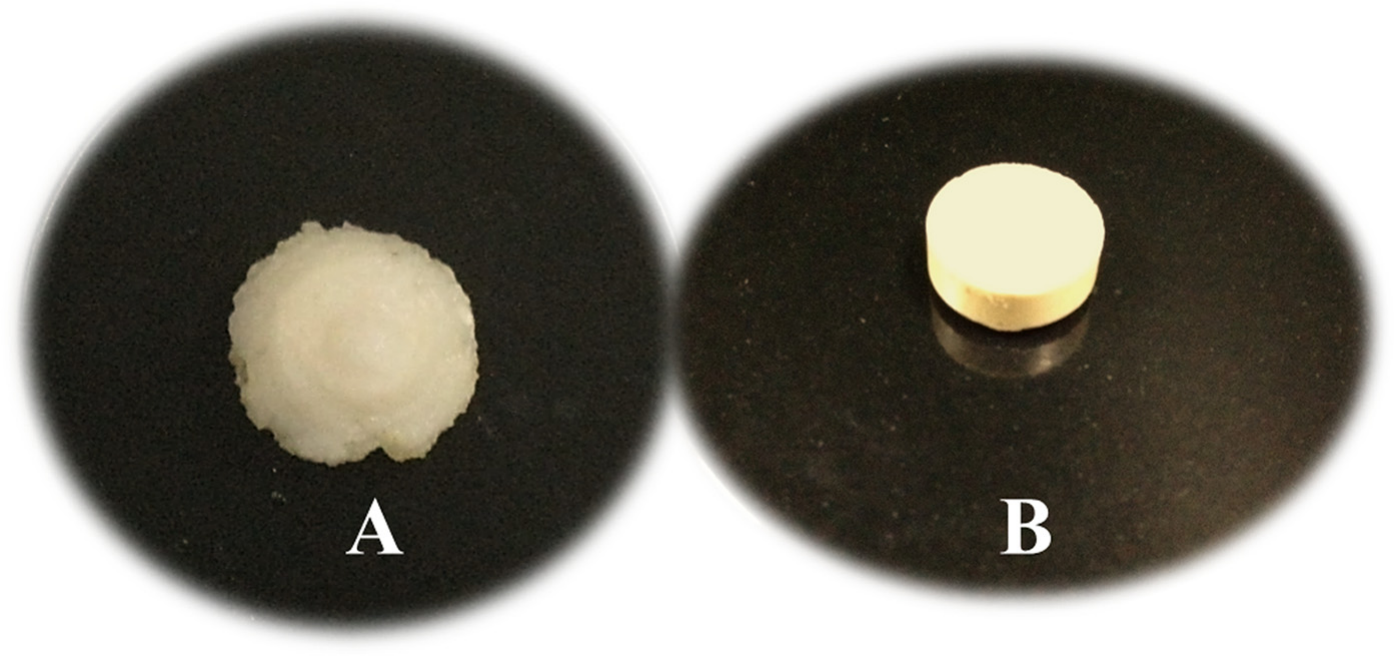
Formulated imatinib mesylate floating sustained-release tablet F6 (B), and swelled tablet after 24 hours (A) in 0.1 N HCl.

As shown in Figs 5 and 3, sufficient swelling behavior was observed in the selected formulated tablet (F6) in terms of proper swelling indices among the time and integrity of tablet after 24 hours.

### Floating behavior

The investigated gastric floating system employed NaHCO_3_ as a gas-forming agent dispersed in a hydrogel matrix. *In vitro* testing revealed that most formulations are able to maintain the drug buoyancy for more than 18 hours (Table 3). It is likely that efficient entrapment of the generated gas bubbles, due to the formation of gel layers by the investigated polymers, may have led to increased tablet porosity, causing it to float for a longer time on the (0.1N HCl) test medium.

**Table 3:** Floating lag time and duration profile of eight different formulated FSR tablets.

Formula code	Floating lag time duration (s)	Total floating time duration (h)
**F1**	118.94 ± 2.43	>24
**F2**	53.34 ± 2.06	18
**F3**	32.73 ± 1.63	>24
**F4**	29.83 ± 2.7	>24
**F5**	31.79 ± 1.22	>24
**F6**	35.54 ± 1.29	>24
**F7**	23.21 ± 1.04	>24
**F8**	24.60 ± 2.04	21

**Fig 6:**
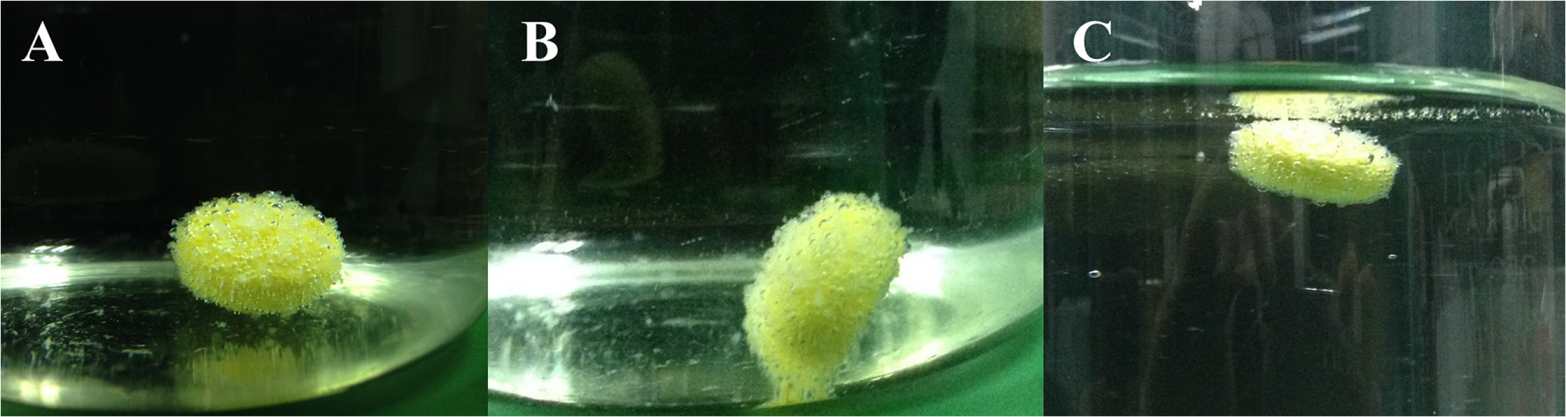
Enclosed effervescent system of the formulated imatinib mesylate (F6) floating sustained-release tablet with carbon dioxide-generated bubbles in 0.1N HCl, (A), after 10 s (B) and after 13 s (C) floated after 36 s.

**Fig 7:**
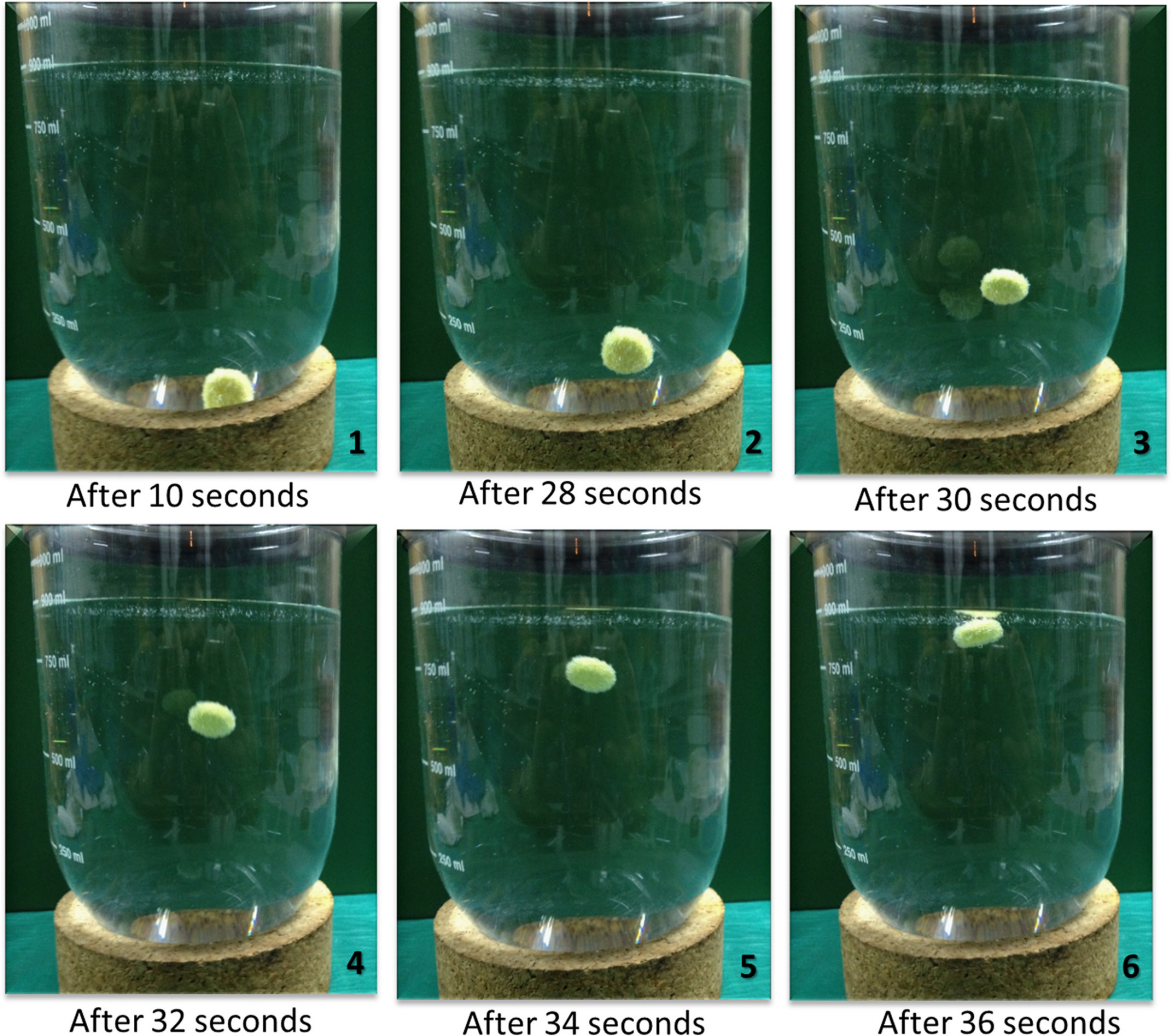
Photographs showing the floating lag time of formula F6 taken during the in vitro floating study in 900 mL 0.1 N HCl at different time intervals.

The fabrication of the matrices is such that, when the tablet arrives in the stomach, the carbon dioxide gas released due to the acidity of the gastric content is trapped within the jellified hydrocolloid [57]. As demonstrated in Figs 6 and 7, carbon dioxide bubbles were generated, making the tablet float. The extended residence time of the drug in the stomach could cause an increase in absorption [58]. This is of particular advantage as the stomach is the essential release site for Imatinib mesylate and then absorb through either stomach or upper part of intestine [59]. One of the factors determining the behavior of the effervescent system is their floating lag time (Fig 7). Maximum floating lag time belonged to F1 with 119s and F8 had minimum floating lag time with 24s. Therefore, the floating lag time for all tablets was found to be below 120s.

As shown in Table 3, formulae F1-F4 consisting of a decrease in HPMC K4M and increased sodium alginate exhibited decreasing floating lag time. This can be explained in relation to the swelling index study, whereby the least amount of HPMC K4M with a high amount of sodium alginate exhibited the highest swelling activity. As swelling increased, more CO_2_ gas was formed by the reaction of sodium bicarbonate with the acidic medium upon penetration of fluid into the tablet matrix. Sodium alginate, which is known to be a more porous hydrogel, is able to entrap and release more CO_2_, producing faster buoyancy. F5-F8, with varying amounts of sodium alginate and constant composition of HPMC K4M, however, did not significantly influence the floating lag time of tablets.

### Drug release study

**Fig 8:**
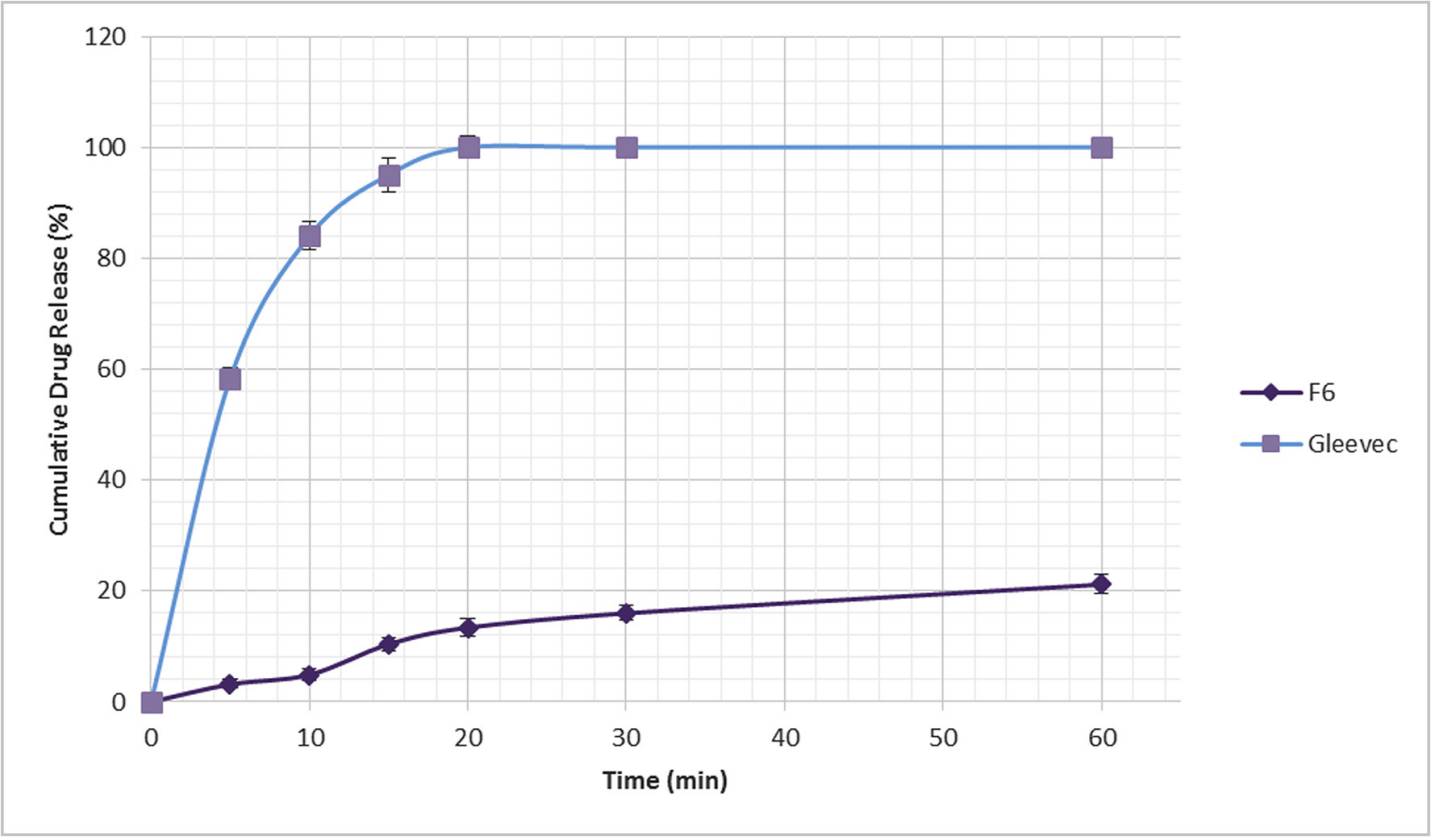
Gleevec vs. FSR-tablet (F6) dissolution profile.

An immediate drug release rate was achieved following the dissolution of Gleevec 400 mg tablets in 0.1 N HCl, where 99.56% of the drug was released within 20 minutes while chosen formulated FSR tablet F6 released only 13.4% after 20 minutes and 99.5% drug release was achieved after approximately 23 hours (Fig 8).

**Fig 9:**
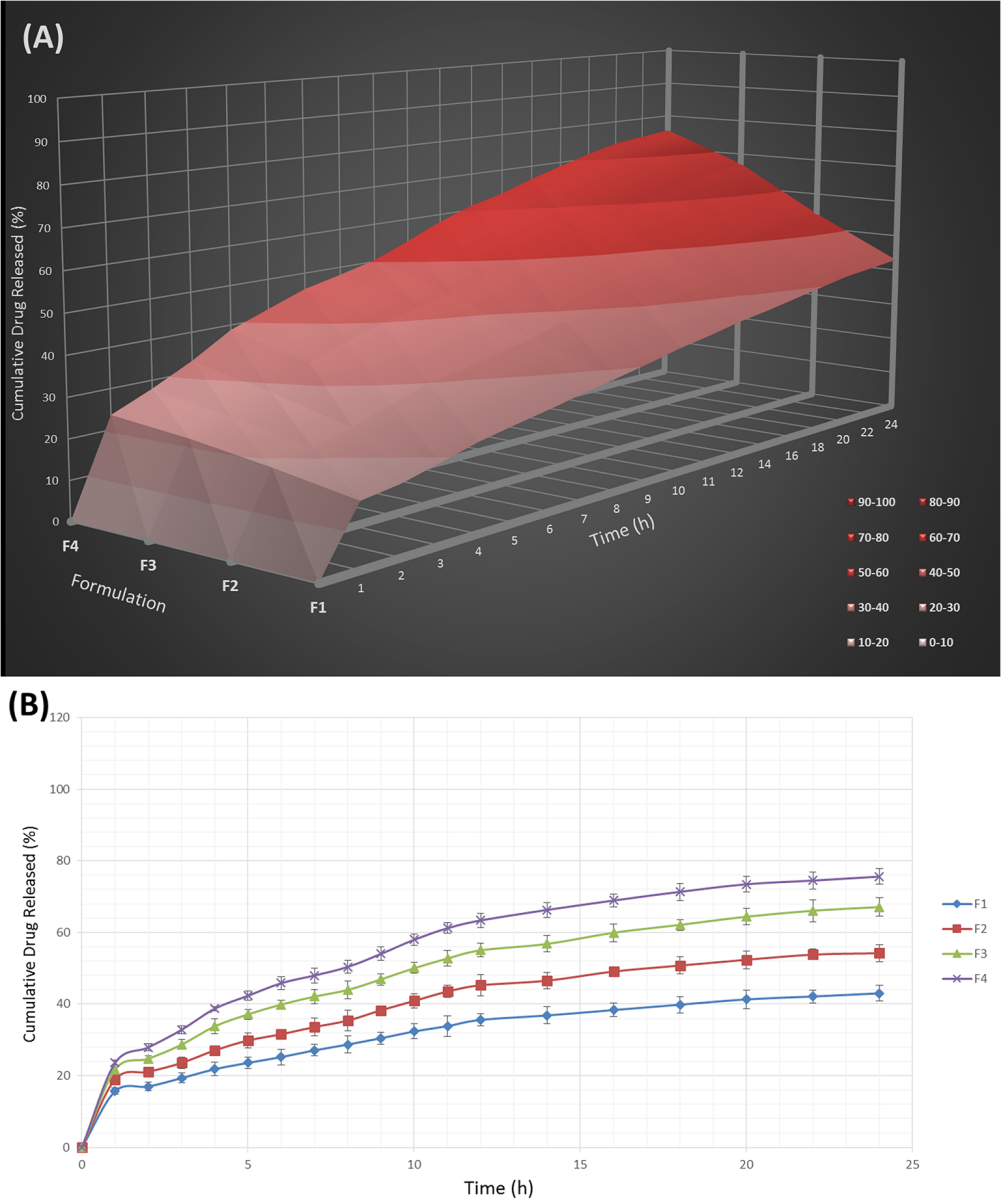
The influence of the HPMC K4M/Na alginate ratio on the release of Imatinib mesylate from the FSR tablets (A) and their release profile (B) in 0.1 N HCl (PH 1.2) at 37± o.5°C (mean ± SD, n = 3).

**Fig 10:**
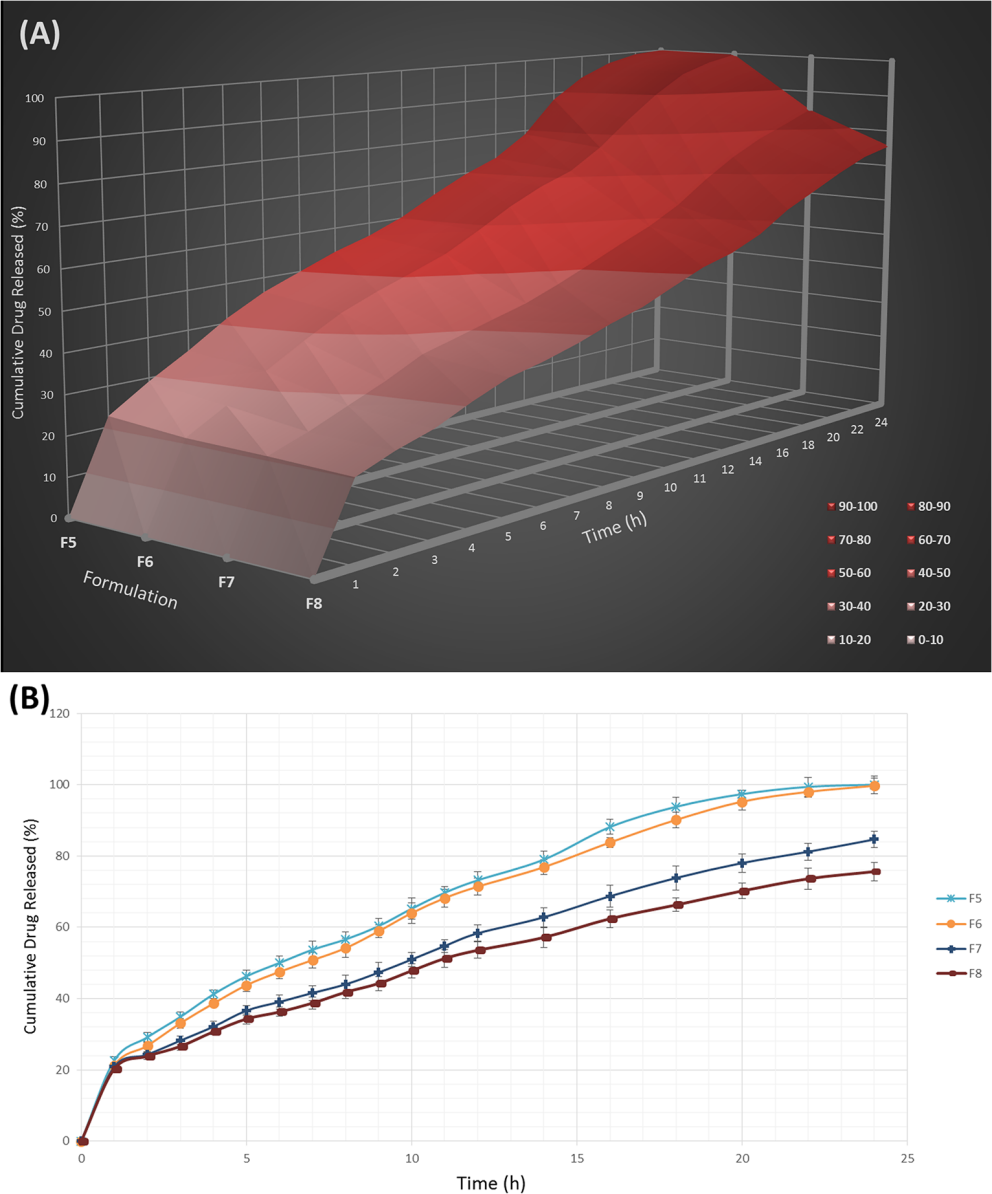
The influence of the Na alginate concentration on the release of Imatinib mesylate from the FSR tablets (A) and their release profile (B) in 0.1 N HCl (PH 1.2) at 37± o.5°C (mean ± SD, n = 3).

**Fig 11:**
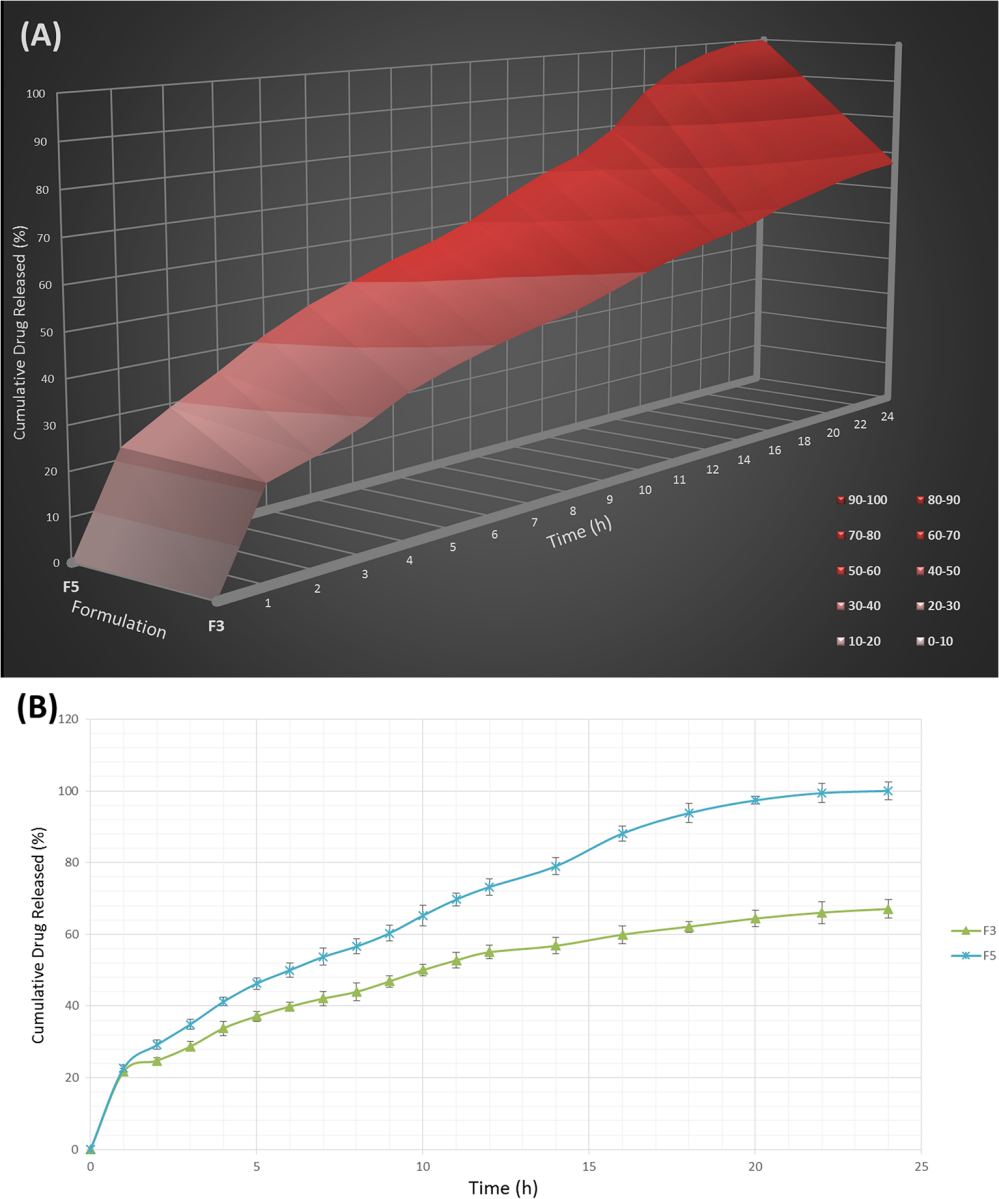
The influence of the Carbomer 934P concentration on the release of Imatinib mesylate from the FSR tablets (A) and their release profile (B) in 0.1 N HCl (PH 1.2) at 37± o.5°C (mean ± SD, n = 3).

By varying the type and concentration of polymer(s) in the current study, diverse drug release profiles were accomplished. The influence of the HPMC K4M / sodium alginate ratio on the release of Imatinib mesylate from the floating sustained release tablets in 0.1 N HCl (pH 1.2) at 37 ± 0.5°C is shown in Fig 9. The higher the amount of HPMC, the slower the rate of drug release. This is due to the increment of gel viscosity upon contact with aqueous fluids, prior to increasing amount of HPMC. However, as the concentration of Na alginate decreased in the tablet matrix (F5-F8) at a constant amount of HPMC, the drug release rate also decreased (Fig 10). This shows that Na alginate was effective in drug release retardation but did not play a major role compared to carbomer. In the case of carbomer, as can be seen in Fig 11, a higher amount of the polymer promotes better retarding behavior. Its volatile characteristic, as it is dispersed in the test medium environment, allows it to expand and reduce the inbound space within the tablet matrices, thus toenailing the control of the release of the drug more efficiently.

It is noteworthy that all formulations did not show burst effects. The burst release of drugs was effectively reduced by the strong surface barriers produced by the polymers upon contact with aqueous media. Taking into consideration that the goal of the study was to achieve a complimentary system of good floating behavior and sustained drug release characteristics, which can be achieved by minimal floating lag time and 24-hour consistent drug release that remains afloat, formula F6 successfully fitted the criteria and was thus chosen for stability studies.

### Drug release kinetics

The results of the release kinetics analyses of Imatinib from the floating sustained-release tablets are as shown in Table 4. The mechanism of release for all the formulations were determined by finding the R^2^ value for each release kinetic model following the Zero order, First order, Higuchi, Hixson-Crowell, Weibull and Korsmeyer-Peppas models. The highest correlation coefficient (R^2^) value, which was derived according to its respective kinetic model, indicated its release pattern.

**Table 4:** Release kinetics correlation coefficient (R^2^) parameter of Imatinib mesylate from the prepared floating sustained-release tablets.

	Korsmeyer-Peppas							
Formula code	R2	Diffusional exponent (n)	Order of release	Zero Order	First Order	Higuchi	Hixson-Crowell	Weibull
**F1**	0.9802	0.3610	Fickian	0.9378	0.9591	0.9881	0.8939	0.8846
**F2**	0.9797	0.3762	Fickian	0.9336	0.9629	0.9850	0.8891	0.8872
**F3**	0.9895	0.3904	Fickian	0.9314	0.9773	0.9893	0.8766	0.8865
**F4**	0.9902	0.4001	Fickian	0.9123	0.9743	0.9818	0.8540	0.8779
**F5**	0.9933	0.5018	Non-Fickian	0.9668	0.8517	0.9942	0.9120	0.9878
**F6**	0.9936	0.5223	Non-Fickian	0.9733	0.8333	0.9948	0.9162	0.9860
**F7**	0.9769	0.4793	Non-Fickian	0.9887	0.9867	0.9882	0.9508	0.9709
**F8**	0.9778	0.4514	Non-Fickian	0.9855	0.9970	0.9911	0.9469	0.9584

Release kinetic analyses indicated that formulations F1-F6 fitted the Higuchi model. This model can be used to describe drug release from different types of modified release dosage forms, particularly matrix tablets with water-soluble drugs. F7 was accorded to fit into the Zero order model, which represents matrix tablets with low-soluble drugs, and F8 complied with the First order model, which describes dosage forms containing water-soluble drugs in porous matrices. Polymeric system mostly releases the drug through diffusion according to the Fickian diffusion. However, when swelling polymers are present in the formulae, such as sodium alginate and/or HPMC K4M, other processes might happen, including water imbibition or relaxation of polymer chains, resulting in polymers swelling and significant volume expansion. Korsmeyer-Peppas equation proposes two evidently independent mechanisms of active component transport in order to justify the release of drug from a swelling polymer; the case-II transport and the Fickian diffusion. F1-F6 indicated high R2 more than 0.99 for the Korsmeyer-Peppas model, which F1-F4 followed the Fickian while F5, F6 adopted the non-Fickian order of release [60].

### Tablet physical stability study

Paired Student’s t-test on the stability studies results for the 6-month showed no significant difference (p > 0.05) in tablet friability, hardness and drug content (Table 5). A significant increase (p < 0.05) in the floating lag time of formula F6, from 35.54 ± 1.29 seconds to 53.00 ± 3.5 seconds after storage for 6 months at 40°C and 75% RH was detected. This could be explained by the reaction that might have occurred between NaHCO_3_ and moisture throughout the storage period, decreasing its initial content of NaHCO_3_, which plays an important role in the buoyancy of tablets [61]. Kuu et al. studied the effect of relative humidity and temperature on moisture absorption and stability of the NaHCO_3_ powder. It was concluded that at 25°C, the powder is recorded to be stable below 76% RH, while at 40°C, the powder is observed to degrade at 75% RH [62]. This shows that the increase in temperature and humidity at equilibrium would result in an increase in the rate of moisture uptake by the tablets.

**Table 5:** Physical/Floating properties of the selected Imatinib mesylate FSR tablet after 6 months at 40°C and 75% RH.

F6 characterization	Fresh tablet	After 6 month
**Floating Lag Time (s)**	35.54 ± 1.29	53.00 ± 3.5
**Total Floating Time (h)**	24.48’ ± 04’	24.53’ ± 06’
**Hardness (N)**	88.68 ± 0.91	86.68 ± 3.4
**Friability (%)**	0.37 ± 0.05	0.36 ± 0.02
**Drug content uniformity (%)**	99.74 ± 0.89	99.37 ± 1.3

### *In vivo* floating result

**Fig 12:**
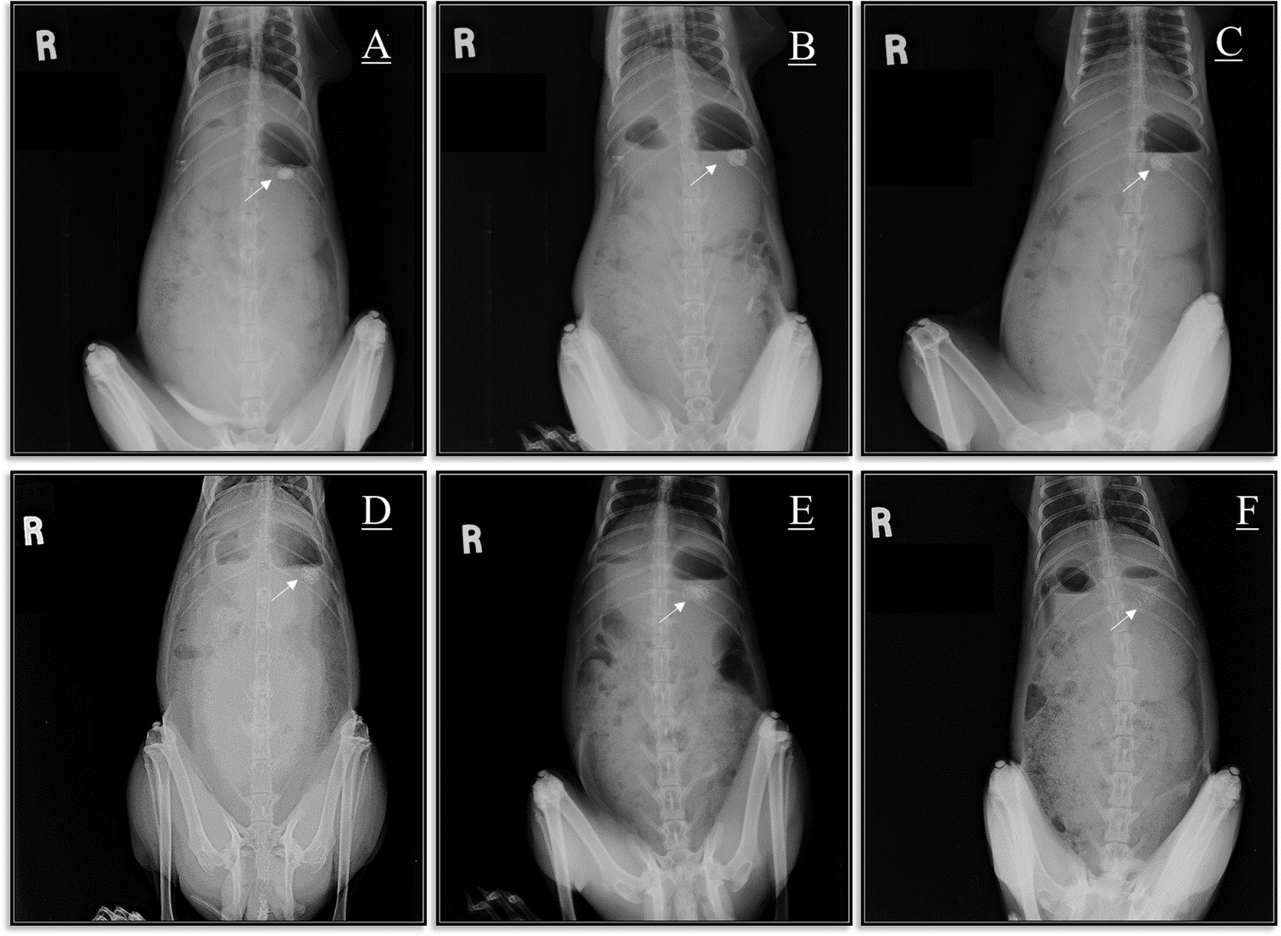
(A–F) X-rays demonstrating the position of the floating barium sulfate-labeled tablet in the gastrointestinal tract of New Zealand rabbits at different time points. X-ray taken at (A) 15 min, (B) 4 h, (C) 8 h, (D) 12 h, (E) 18 h, and (F) 24 h.

As expected, the 350 mg barium sulfate-loaded tablet’s *in vitro* total floating time was slightly more than 24 h, which was shorter than the floating time of the original formulation (F6). Floating lag time also was increased to 58 seconds which is still in sufficient range. This is because BaSO4 has a higher density of 4.5 g/cm^3^, compared to imatinib mesylate with a density of 1.25 g/cm^3^. Fig 12 shows the X-ray images of the barium sulfate-loaded tablets, which were floating in the New Zealand rabbits’ stomachs. The floating barium sulfate-loaded tablets were observed to remain buoyant in the stomach for 24 h. These results ensured that the formulated HPMC-SA-CP hydrophilic matrix, with Na bicarbonate as an effervescent agent, can provide acceptable 24-hour gastroretentive behavior *in vivo*. As demonstrated in Fig 12, during the experiment, the tablet’s matrix complex slowly swelled in the stomach but still stayed integrated after 24 hours.

### *In vivo* release study

**Fig 13:**
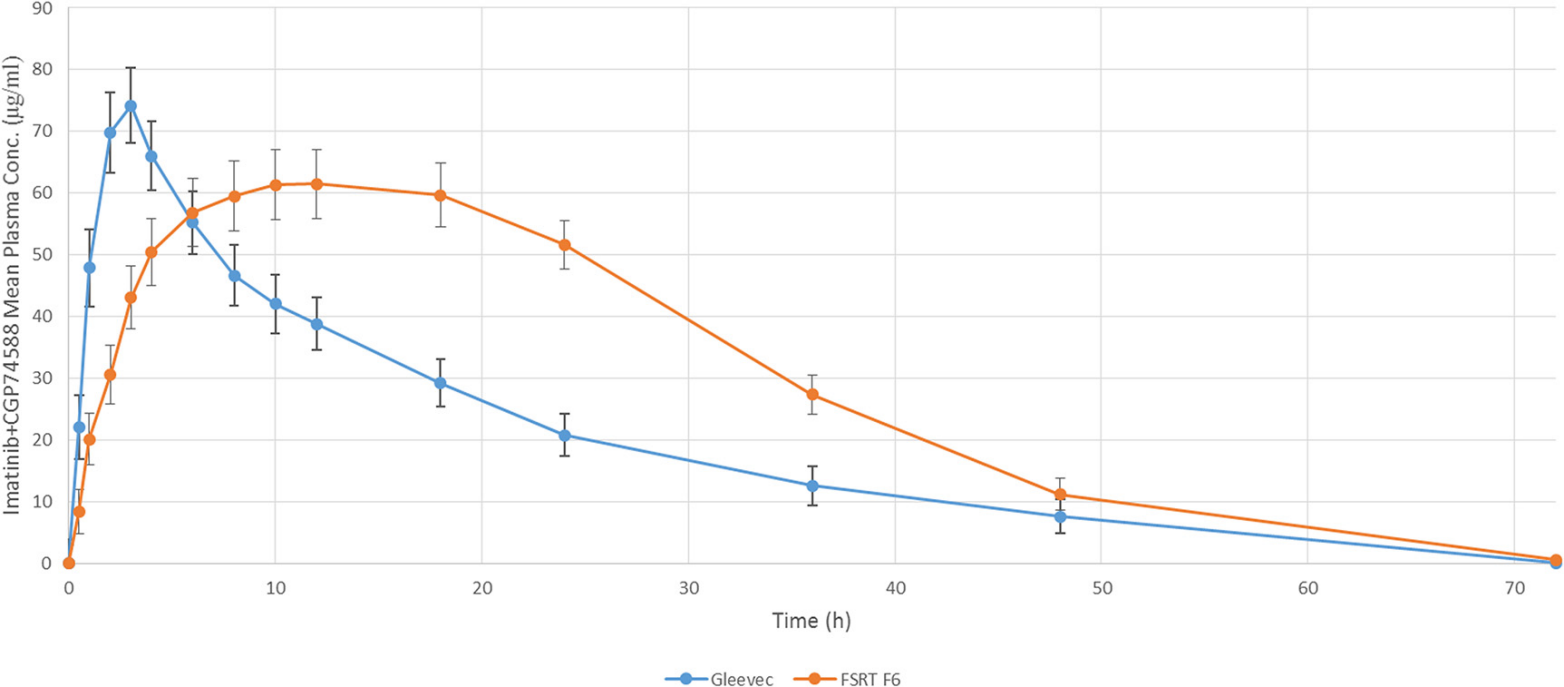
Mean plasma concentration ± SD profile of imatinib+ CGP74588 after 200 mg oral administration of Gleevec or formulated FSR tablet (F6) in 12 healthy rabbits.

**Fig 14:**
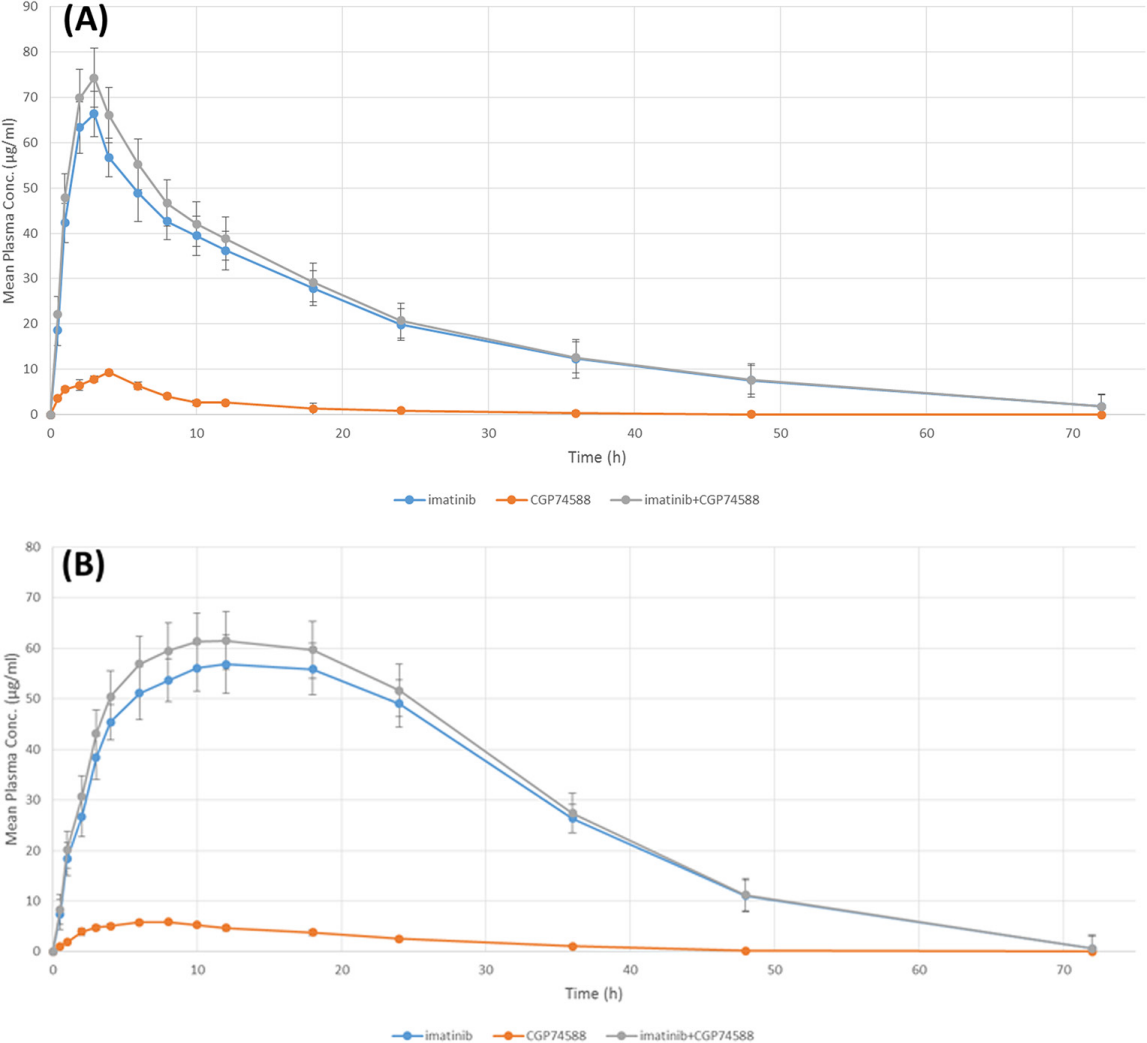
Mean plasma concentration profile of imatinib and its major metabolite CGP74588 after 200 mg oral administration of (A) Gleevec or (B) formulated FSR tablet (F6) in healthy rabbits (mean ± SD, n = 6).

Rabbits were administered with 200 mg Imatinib in commercial (Gleevec) or formulated Floating-sustained-release tablet (F6) form. Descriptional pharmacokinetic mass-balance study revealed that after oral administration, Imatinib is promptly absorbed in both Gleevec and FSR formulated tablets. The mean peak plasma concentration of Imatinib (C_max_) after a single oral dose of 200 mg was detected to be 74.235 and 61.524 (**μ**g/ml) for the Gleevec and the formulated FSR tablet, respectively. C_max_ was achieved after 2 hours and 50 minutes with the Gleevec, while with the formulated FSR tablet, t_max_ was 11.20′ h. Therefore, it can be concluded that the Imatinib C_max_ released from F6 was 12.711 (**μ**g/ml) less compared to the Gleevec. This could prevent the high dosage toxicity of Imatinib [63]. Moreover, longer t_max_ and t_1/2_ caused by the steadier plasma concentration improved the Imatinib efficiency through F6 sustained release (Fig 13). CGP74588 as a major active metabolite of Imatinib was detected in blood plasma [64]. As demonstrated in Fig 14, the highest concentrations of CGP74588 in blood were around 14.1% and 10.3% of unchanged Imatinib in Gleevec and F6, respectively.

The pharmacokinetic characteristics of Imatinib and its major metabolit which were released from Gleevec and F6, investigated in twelve healthy rabbits, are demonstrated in Table 6.

**Table 6:** Pharmacokinetic parameters of Gleevic and formulated Imatinib FSR (F6) tablets after oral administration of 200 mg (mean ± SD, n = 6).

	Imatinib mesylate 200 mg (oral dose)	
PK parameter	Gleevec	F6 SR formulated tablet
**C**_**max**_ **(μg/ml)**	74.235 ± 6.511	61.524 ± 5.673
**t**_**max**_ **(h)**	2.50′ ± 0.30′	11.20′ ± 1.10′
**t**_**1/2**_ **(h)**	14.30′ ± 1.20′	34.20′ ± 3
**AUC**_**(0–24)**_ **(h·μg/ml)**	970.952 ± 173.406	1283.233 ± 238.829
**AUC**_**(0-t)**_ **(h·μg/ml)**	1406.407 ± 216.633	2131.293 ± 401.182
**AUC**_**(0-∞)**_ **(h·μg/ml)**	1444.528 ± 195.073	2145.493 ± 394.537

## Conclusion

A promising floating sustained-release Imatinib mesylate tablet was successfully formulated with HPMC-SA-CP hydrophilic matrix using the effervescent technique. Tablets containing HPMC K4M (14.67%, w/w), Na alginate (10.67%, w/w), carbomer 934P (1.33%, w/w) and NaHCO3 (9.33%, w/w) (formula F6) showed satisfactory results with respect to physical characterization, sustained drug release rates, floating lag time, total floating duration and swelling behavior. Moreover, Formula F6 showed acceptable physical stability when stored at 40°C under 75% RH for 6 months. The optimized formulation (F6) had gastroretentive ability *in vivo* and therefore, the formulated floating sustained-release tablet is a feasible approach for a sustained-release preparation of imatinib mesylate, which has a high absorption rate in the stomach or in the upper part of the gastrointestinal tract. Furthermore, according to *in vivo* release study, the even and prolonged absorption phase associate with preservation of plasma concentration for extended time following administration of formulated FSR tablet offer the lower chance of dose-dependent side effects and improve the imatinib efficiency.
