# Correction: Formulation and *In Vitro*, *In Vivo* Evaluation of Effervescent Floating Sustained-Release Imatinib Mesylate Tablet

**DOI:** 10.1371/journal.pone.0275144

**Published:** 2022-09-21

**Authors:** The PLOS ONE Editors

Following publication, questions were raised about some of the citations and referencing in the published article [1], and it was noted the primary data underlying results in this article were not included with the published article, despite the article’s Data Availability Statement indicating that all relevant data are within the paper. The corresponding author has provided the underlying data set, included with this notice as Supporting Information S1–S7 Files.

The *PLOS ONE* Editors have carried out an assessment of the citation and reference issues raised in consultation with a member of the Editorial Board, who noted that a number of in-text citations appear to be misplaced or incomplete, including the following:

- In the Introduction, the sentence “Its adverse effects include hair loss, diarrhea, nausea, loss of appetite, vomiting, dry skin, muscle cramps and swelling (especially in the legs or around the eyes) [14].” does not appear to include a reference specifically mentioning hair loss, loss of appetite, vomiting, or dry skin.
- In the Drug content uniformity subsection of the Materials and Methods, reference 30 appears to be incorrectly placed.
- In the Friability study subsection of the Materials and Methods, references 30 and 31 appear to be incorrectly placed.
- In the Tablet swelling behaviour subsection of the Materials and Methods, the first sentence states ‘The method by Dorozynski et al. was used to determine the tablets’ swelling behavior in this study, which was done in triplicates [32].” In the Reference list, Dorozynski et al. is numbered as reference 33.
- In the *In vitro* dissolution study subsection of the Materials and Methods, reference 40 appears to be incorrectly placed.
- Reference (Tadros, 2010) is included three times as references 8, 27 and 44 in article [1].

## Supporting information

S1 File Underlying data supporting the Imatinib swelling charts in Figures 2–4. (PDF)

S2 File Underlying data supporting the Imatinib mesylate release charts in Figures 9–11. (PDF)

S3 File Underlying data supporting the charts in Figures 13–14. (PDF)

S4 File Underlying data supporting the charts in Figures 13–14. (PDF)

S5 File Underlying data supporting the results in Table 4. (PDF)

S6 File Underlying data supporting the results in Table 4. (PDF)

S7 File Underlying data supporting the results in Table 4. (PDF)
