# Supplementary figures and images for: Correction: Formulation and In Vitro, In Vivo Evaluation of Effervescent Floating Sustained-Release Imatinib Mesylate Tablet
Source: PLoS One. 2022 Sep 21;17(9):e0275144. doi: 10.1371/journal.pone.0275144 (PMC9491528; doi:10.1371/journal.pone.0275144)

|    |   |      |      |      |      |      |      |      |       |
|----|---|------|------|------|------|------|------|------|-------|
|    | 0 | 3    | 6    | 9    | 12   | 15   | 18   | 21   | 24    |
| F1 | 0 | 2.5  | 3.2  | 3.6  | 4    | 4.2  | 3.6  | 2.7  | 2.2   |
| F2 | 0 | 2.5  | 3.3  | 3.8  | 4.3  | 4.5  | 3.9  | 3.1  | 2.6   |
| F3 | 0 | 2.6  | 3.5  | 4.1  | 4.5  | 4.7  | 4.2  | 3.4  | 2.9   |
| F4 | 0 | 3    | 4.1  | 4.6  | 4.9  | 4.9  | 4.6  | 4.2  | 3.7   |
| 1  | 0 | 0.09 | 0.1  | 0.12 | 0.21 | 0.24 | 0.25 | 0.1  | 0.05  |
| 2  | 0 | 0.15 | 0.1  | 0.18 | 0.25 | 0.27 | 0.26 | 0.21 | 0.13  |
| 3  | 0 | 0.07 | 0.1  | 0.2  | 0.24 | 0.27 | 0.3  | 0.3  | 0.07  |
| 4  | 0 | 0.1  | 0.17 | 0.14 | 0.2  | 0.22 | 0.28 | 0.17 | 0.012 |

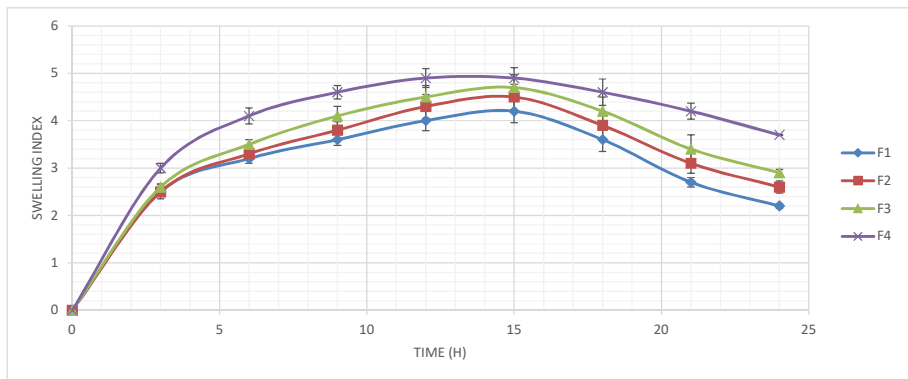

|    |   |      |      |      |      |      |      |     |      |
|----|---|------|------|------|------|------|------|-----|------|
|    | 0 | 3    | 6    | 9    | 12   | 15   | 18   | 21  | 24   |
| F3 | 0 | 2.6  | 3.5  | 4.1  | 4.5  | 4.7  | 4.2  | 3.4 | 2.9  |
| F5 | 0 | 2.2  | 3    | 3.5  | 3.8  | 4    | 3.5  | 2.9 | 2.3  |
| 3  | 0 | 0.07 | 0.1  | 0.2  | 0.24 | 0.27 | 0.3  | 0.3 | 0.07 |
| 5  | 0 | 0.12 | 0.21 | 0.18 | 0.23 | 0.28 | 0.17 | 0.1 | 0.08 |

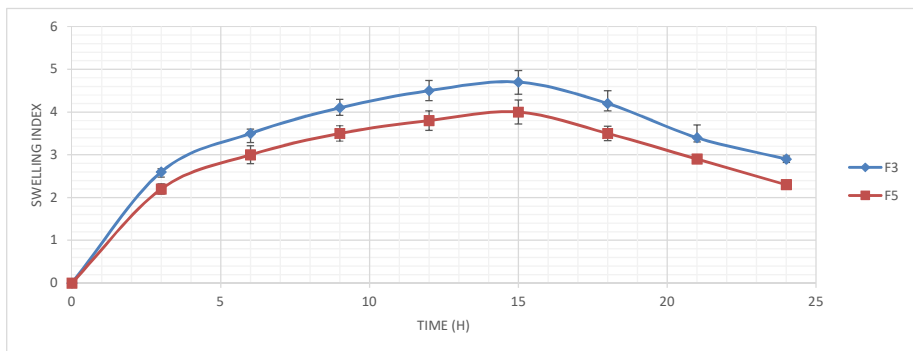

|    |   |      |      |      |      |      |      |      |      |
|----|---|------|------|------|------|------|------|------|------|
|    | 0 | 2.2  | 3    | 3.5  | 3.8  | 4    | 3.5  | 2.9  | 2.3  |
| F5 | 0 | 2    | 2.8  | 3.3  | 3.7  | 3.8  | 3.2  | 2.6  | 1.9  |
| F6 | 0 | 1.8  | 2.6  | 3.1  | 3.5  | 3.6  | 3.1  | 2.4  | 1.7  |
| F7 | 0 | 1.5  | 2.4  | 2.8  | 3.2  | 3.3  | 2.7  | 2.1  | 1.4  |
| F8 | 0 | 0.12 | 0.21 | 0.18 | 0.23 | 0.28 | 0.17 | 0.1  | 0.08 |
| 6  | 0 | 0.06 | 0.17 | 0.11 | 0.18 | 0.21 | 0.2  | 0.13 | 0.07 |
| 7  | 0 | 0.03 | 0.1  | 0.2  | 0.3  | 0.31 | 0.22 | 0.16 | 0.09 |
| 8  | 0 | 0.1  | 0.13 | 0.18 | 0.21 | 0.23 | 0.2  | 0.2  | 0.1  |

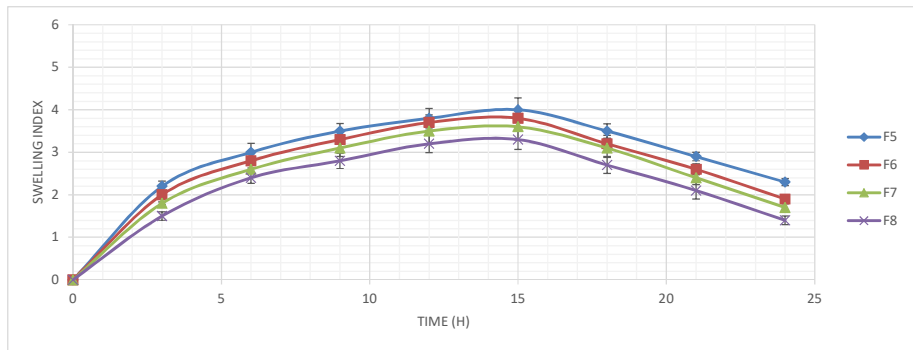

Supplement: S1 File — (PDF) [file pone.0275144.s001.pdf]

|    |   |       |       |       |       |       |       |       |       |       |       |       |       |       |       |       |       |       |       |
|----|---|-------|-------|-------|-------|-------|-------|-------|-------|-------|-------|-------|-------|-------|-------|-------|-------|-------|-------|
|    | 0 | 1     | 2     | 3     | 4     | 5     | 6     | 7     | 8     | 9     | 10    | 11    | 12    | 14    | 16    | 18    | 20    | 22    | 24    |
| F1 | 0 | 15.67 | 17    | 19.35 | 21.84 | 23.6  | 25.23 | 27.1  | 28.72 | 30.48 | 32.4  | 33.85 | 35.6  | 36.85 | 38.35 | 39.85 | 41.32 | 42.13 | 43.01 |
| F2 | 0 | 19    | 21.11 | 23.6  | 27.1  | 29.85 | 31.6  | 33.62 | 35.41 | 38.29 | 40.9  | 43.51 | 45.25 | 46.55 | 49.1  | 50.77 | 52.38 | 53.81 | 54.2  |
| F3 | 0 | 21.75 | 24.76 | 28.7  | 33.81 | 37.16 | 39.88 | 42.1  | 43.96 | 46.91 | 50.03 | 52.73 | 55.02 | 56.81 | 59.93 | 62.13 | 64.41 | 66.07 | 67.1  |
| F4 | 0 | 23.59 | 27.91 | 32.85 | 38.74 | 42.34 | 45.85 | 48    | 50.38 | 54.02 | 57.98 | 61.18 | 63.35 | 66.3  | 68.92 | 71.35 | 73.47 | 74.49 | 75.6  |
| F5 | 0 | 22.67 | 29.16 | 34.88 | 41.21 | 46.27 | 50.02 | 53.72 | 56.6  | 60.34 | 65.24 | 69.7  | 73.21 | 79    | 88.13 | 93.81 | 97.37 | 99.41 | 100   |
| F6 | 0 | 21.19 | 26.78 | 33.1  | 38.64 | 43.77 | 47.52 | 50.8  | 54.17 | 58.9  | 64    | 68.2  | 71.42 | 76.88 | 83.81 | 90.13 | 95.26 | 98    | 99.71 |
| F7 | 0 | 20.88 | 24.27 | 28.22 | 32.13 | 36.64 | 39.06 | 41.58 | 44    | 47.31 | 50.97 | 54.76 | 58.32 | 62.82 | 68.72 | 73.84 | 78.03 | 81.2  | 84.65 |
| F8 | 0 | 20.26 | 23.86 | 26.61 | 30.72 | 34.3  | 36.26 | 38.74 | 41.79 | 44.25 | 47.81 | 51.26 | 53.56 | 57.16 | 62.41 | 66.27 | 70.21 | 73.62 | 76.45 |

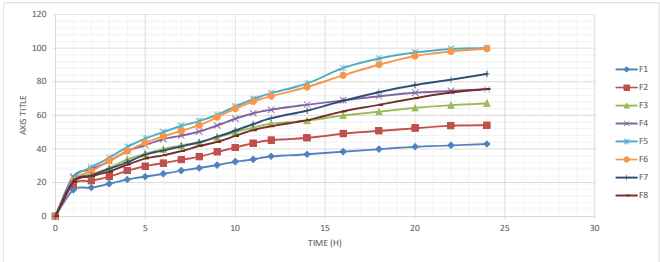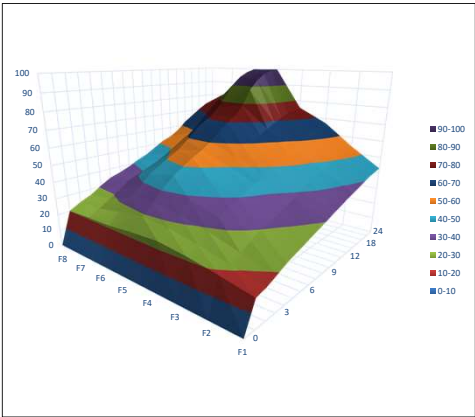

|    |   |       |       |       |       |       |       |       |       |       |       |       |       |       |       |       |       |       |       |
|----|---|-------|-------|-------|-------|-------|-------|-------|-------|-------|-------|-------|-------|-------|-------|-------|-------|-------|-------|
|    | 0 | 1     | 2     | 3     | 4     | 5     | 6     | 7     | 8     | 9     | 10    | 11    | 12    | 14    | 16    | 18    | 20    | 22    | 24    |
| F8 | 0 | 20.26 | 23.86 | 26.61 | 30.72 | 34.3  | 36.26 | 38.74 | 41.79 | 44.25 | 47.81 | 51.26 | 53.56 | 57.16 | 62.41 | 66.27 | 70.21 | 73.62 | 76.45 |
| F7 | 0 | 20.88 | 24.27 | 28.22 | 32.13 | 36.64 | 39.06 | 41.58 | 44    | 47.31 | 50.97 | 54.76 | 58.32 | 62.82 | 68.72 | 73.84 | 78.03 | 81.2  | 84.65 |
| F6 | 0 | 21.19 | 26.78 | 33.1  | 38.64 | 43.77 | 47.52 | 50.8  | 54.17 | 58.9  | 64    | 68.2  | 71.42 | 76.88 | 83.81 | 90.13 | 95.26 | 98    | 99.71 |
| F5 | 0 | 22.67 | 29.16 | 34.88 | 41.21 | 46.27 | 50.02 | 53.72 | 56.6  | 60.34 | 65.24 | 69.7  | 73.21 | 79    | 88.13 | 93.81 | 97.37 | 99.41 | 100   |

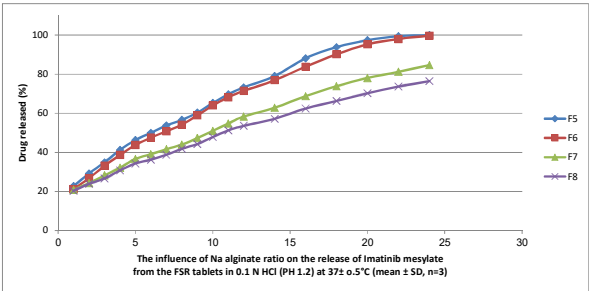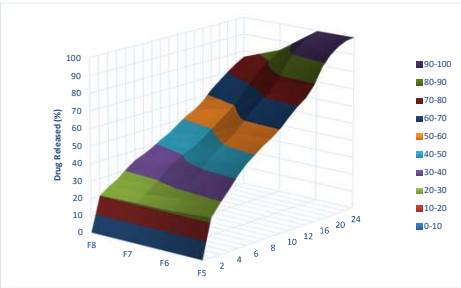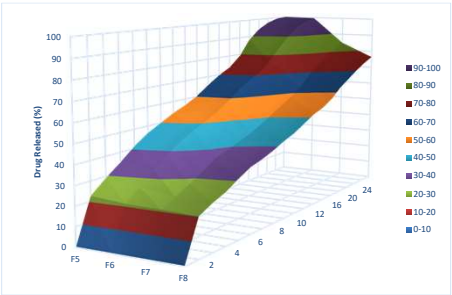

|    |       |       |       |       |       |       |       |       |       |       |       |       |       |       |       |       |       |       |
|----|-------|-------|-------|-------|-------|-------|-------|-------|-------|-------|-------|-------|-------|-------|-------|-------|-------|-------|
|    | 1     | 2     | 3     | 4     | 5     | 6     | 7     | 8     | 9     | 10    | 11    | 12    | 14    | 16    | 18    | 20    | 22    | 24    |
| F1 | 15.67 | 17    | 19.35 | 21.84 | 23.6  | 25.23 | 27.1  | 28.72 | 30.48 | 32.4  | 33.85 | 35.6  | 36.85 | 38.35 | 39.85 | 41.32 | 42.13 | 43.01 |
| F2 | 19    | 21.11 | 23.6  | 27.1  | 29.85 | 31.6  | 33.62 | 35.41 | 38.29 | 40.9  | 43.51 | 45.25 | 46.55 | 49.1  | 50.77 | 52.38 | 53.81 | 54.2  |
| F3 | 21.75 | 24.76 | 28.7  | 33.81 | 37.16 | 39.88 | 42.1  | 43.96 | 46.91 | 50.03 | 52.73 | 55.02 | 56.81 | 59.93 | 62.13 | 64.41 | 66.07 | 67.1  |
| F4 | 23.59 | 27.91 | 32.85 | 38.74 | 42.34 | 45.85 | 48    | 50.38 | 54.02 | 57.98 | 61.18 | 63.35 | 66.3  | 68.92 | 71.35 | 73.47 | 74.49 | 75.6  |

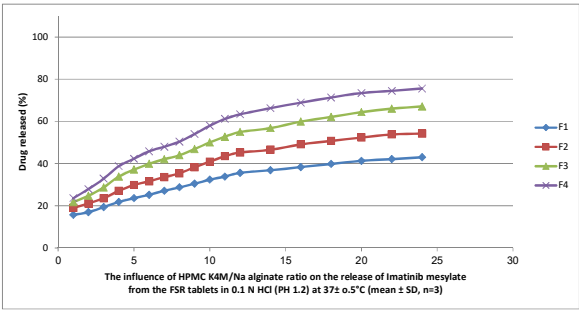

Supplement: S2 File — (PDF) [file pone.0275144.s002.pdf]

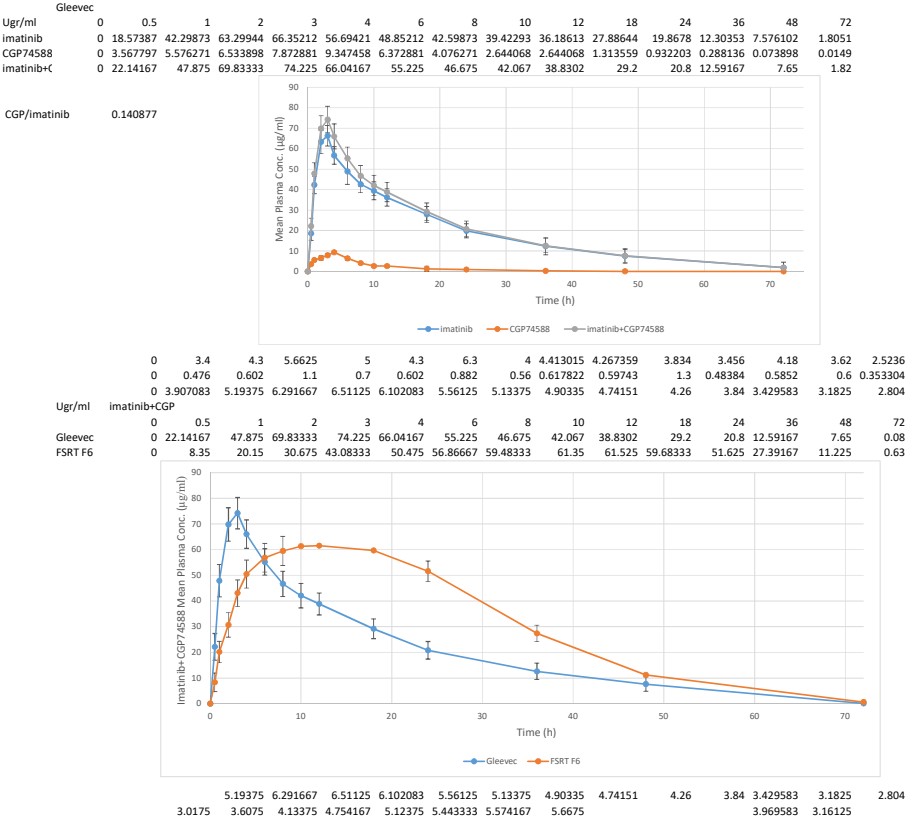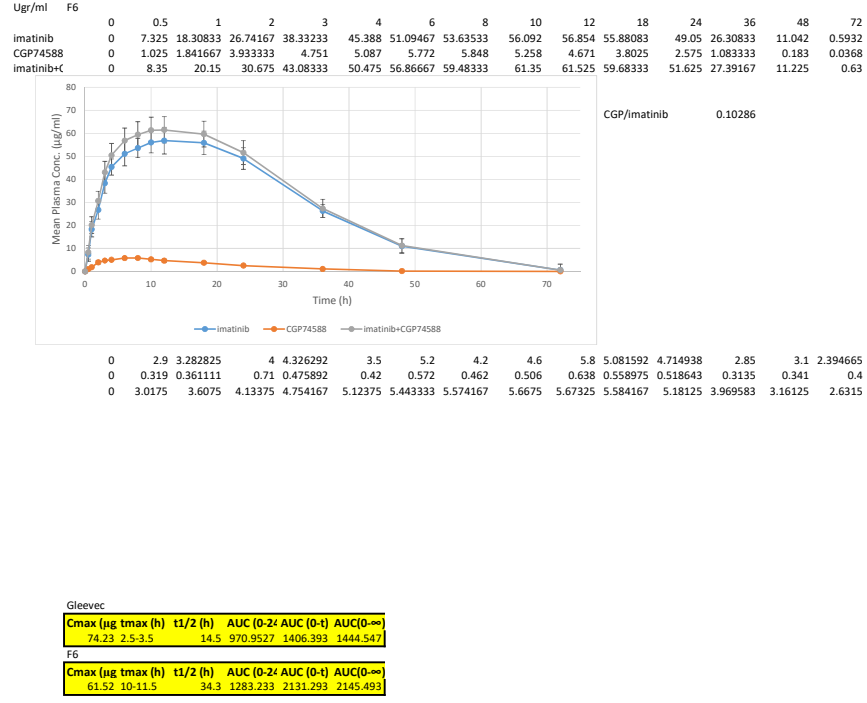

Supplement: S4 File — (PDF) [file pone.0275144.s004.pdf]

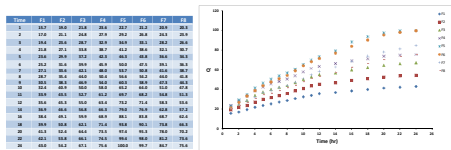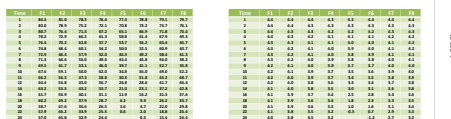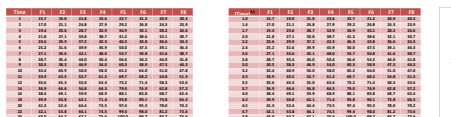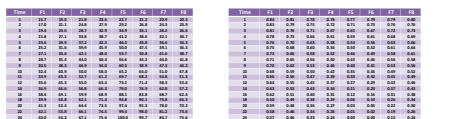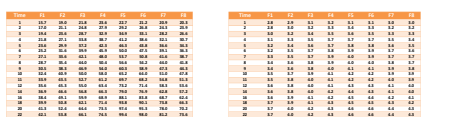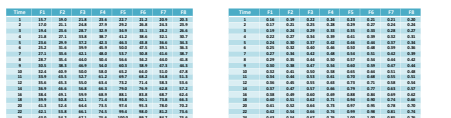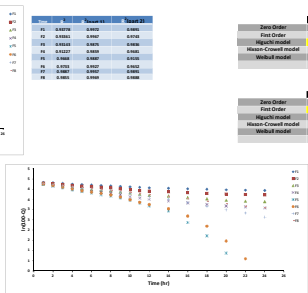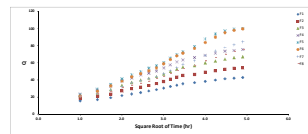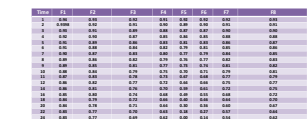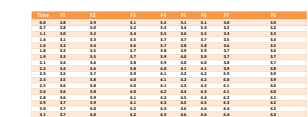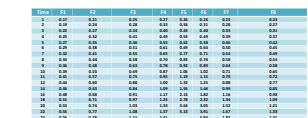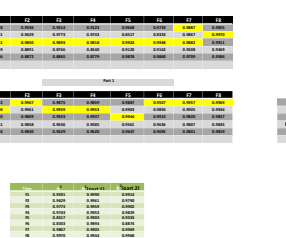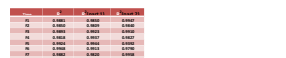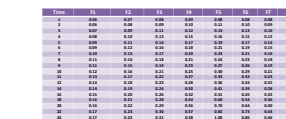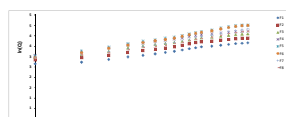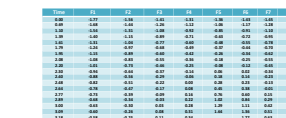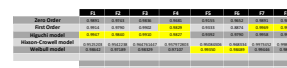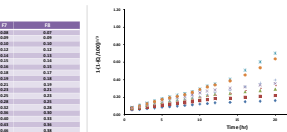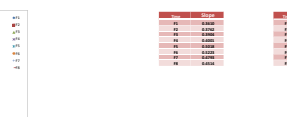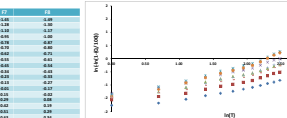

Supplement: S6 File — (PDF) [file pone.0275144.s006.pdf]
