# Supplementary material for: Correction: Formulation and In Vitro, In Vivo Evaluation of Effervescent Floating Sustained-Release Imatinib Mesylate Tablet
Source: PLoS One. 2022 Sep 21;17(9):e0275144. doi: 10.1371/journal.pone.0275144 (PMC9491528; doi:10.1371/journal.pone.0275144)
Supplement: S5 File — (PDF) [file pone.0275144.s005.pdf]

Zero Order

Time	F1	F2	F3	F4	Column1	Column2	Column3	Column4
1	15.7	19.0	21.8	23.6				
2	17.0	21.1	24.8	27.9				
3	19.4	23.6	28.7	32.9				
4	21.8	27.1	33.8	38.7				
5	23.6	29.9	37.2	42.3				
6	25.2	31.6	39.9	45.9				
7	27.1	33.6	42.1	48.0				
8	28.7	35.4	44.0	50.4				
9	30.5	38.3	46.9	54.0				
10	32.4	40.9	50.0	58.0				
11	33.9	43.5	52.7	61.2				
12	35.6	45.3	55.0	63.4				
14	36.9	46.6	56.8	66.3				
16	38.4	49.1	59.9	68.9				
18	39.9	50.8	62.1	71.4				
20	41.3	52.4	64.4	73.5				
22	42.1	53.8	66.1	74.5				
24	43.0	54.2	67.1	75.6				

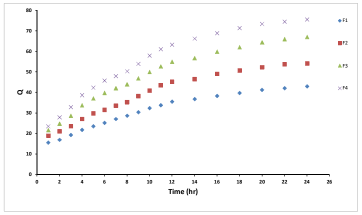

Time	R^2	R^2(part 1)	R^2(part 2)
F1			
F2			
F3			
F4			
F5	0.9378	0.9972	0.9891
F6	0.9336	0.9967	0.9743
F7	0.9314	0.9875	0.9836
F8	0.9123	0.9859	0.9681

	F1	F2	F3	F4
Zero Order	0.9378	0.9336	0.9314	0.9123
First Order	0.9591	0.9629	0.9773	0.9743
Higuchi model	0.9881	0.9850	0.9893	0.9818
Hixson-Crowell model	0.8939	0.8891	0.8766	0.8540
Weibull model	0.8980	0.8872	0.8865	0.8779

Part 1

	F1	F2	F3	F4	Column1	Column2	Column3	Column4
Zero Order	0.9972	0.9967	0.9875	0.9859				
First Order	0.9990	0.9961	0.9959	0.9953				
Higuchi model	0.9850	0.9809	0.9923	0.9937				
Hixson-Crowell model	0.9851	0.9858	0.9636	0.9585				
Weibull model	0.9804	0.9830	0.9629	0.9620				

Part II

	F1	F2	F3	F4
Zero Order	0.9891	0.9743	0.9836	0.9681
First Order	0.9914	0.9790	0.9902	0.9829
Higuchi model	0.9947	0.9840	0.9910	0.9827
Hixson-Crowell model	0.9863	0.9706	0.9799	0.9629
Weibull model	0.99289	0.97189	0.98329	0.97107

First Order

Time	F1	F2	F3	F4
1	84.3	81.0	78.3	76.4
2	83.0	78.9	75.2	72.1
3	80.7	76.4	71.3	67.2
4	78.2	72.9	66.2	61.3
5	76.4	70.2	62.8	57.7
6	74.8	68.4	60.1	54.2
7	72.9	66.4	57.9	52.0
8	71.3	64.6	56.0	49.6
9	69.5	61.7	53.1	46.0
10	67.6	59.1	50.0	42.0
11	66.2	56.5	47.3	38.8
12	64.4	54.8	45.0	36.7
14	63.2	53.5	43.2	33.7
16	61.7	50.9	40.1	31.1
18	60.2	49.2	37.9	28.7
20	58.7	47.6	35.6	26.5
22	57.9	46.2	33.9	25.5
24	57.0	45.8	32.9	24.4

Time	F1	F2	F3	F4
1	4.4	4.4	4.4	4.3
2	4.4	4.4	4.3	4.3
3	4.4	4.3	4.3	4.2
4	4.4	4.3	4.2	4.1
5	4.3	4.3	4.1	4.1
6	4.3	4.2	4.1	4.0
7	4.3	4.2	4.1	4.0
8	4.3	4.2	4.0	3.9
9	4.2	4.1	4.0	3.8
10	4.2	4.1	3.9	3.7
11	4.2	4.0	3.9	3.7
12	4.2	4.0	3.8	3.6
14	4.1	4.0	3.8	3.5
16	4.1	3.9	3.7	3.4
18	4.1	3.9	3.6	3.4
20	4.1	3.9	3.6	3.3
22	4.1	3.8	3.5	3.2
24	4.0	3.8	3.5	3.2

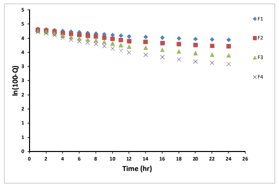

Time	R^2	R^2(part 1)	R^2(part 2)
F5	0.9591	0.9990	0.9914
F6	0.9629	0.9961	0.9790
F7	0.9773	0.9959	0.9902
F8	0.9743	0.9953	0.9829

Higuchi model

Time	F1	F2	F3	F4
1	15.7	19.0	21.8	23.6
2	17.0	21.1	24.8	27.9
3	19.4	23.6	28.7	32.9
4	21.8	27.1	33.8	38.7
5	23.6	29.9	37.2	42.3
6	25.2	31.6	39.9	45.9
7	27.1	33.6	42.1	48.0
8	28.7	35.4	44.0	50.4
9	30.5	38.3	46.9	54.0
10	32.4	40.9	50.0	58.0
11	33.9	43.5	52.7	61.2
12	35.6	45.3	55.0	63.4
14	36.9	46.6	56.8	66.3
16	38.4	49.1	59.9	68.9
18	39.9	50.8	62.1	71.4
20	41.3	52.4	64.4	73.5
22	42.1	53.8	66.1	74.5
24	43.0	54.2	67.1	75.6

$(Time)^{0.}$	F1	F2	F3	F4
1.0	15.7	19.0	21.8	23.6
1.4	17.0	21.1	24.8	27.9
1.7	19.4	23.6	28.7	32.9
2.0	21.8	27.1	33.8	38.7
2.2	23.6	29.9	37.2	42.3
2.4	25.2	31.6	39.9	45.9
2.6	27.1	33.6	42.1	48.0
2.8	28.7	35.4	44.0	50.4
3.0	30.5	38.3	46.9	54.0
3.2	32.4	40.9	50.0	58.0
3.3	33.9	43.5	52.7	61.2
3.5	35.6	45.3	55.0	63.4
3.7	36.9	46.6	56.8	66.3
4.0	38.4	49.1	59.9	68.9
4.2	39.9	50.8	62.1	71.4
4.5	41.3	52.4	64.4	73.5
4.7	42.1	53.8	66.1	74.5
4.9	43.0	54.2	67.1	75.6

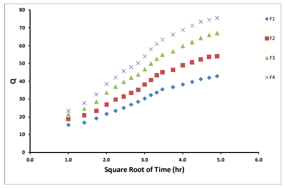

Time	R^2	R^2(part 1)	R^2(part 2)
F5	0.9881	0.9850	0.9947
F6	0.9850	0.9809	0.9840
F7	0.9893	0.9923	0.9910
F8	0.9818	0.9937	0.9827

Hixson-Crowell mode

Time	F1	F2	F3	F4
1	15.7	19.0	21.8	23.6
2	17.0	21.1	24.8	27.9
3	19.4	23.6	28.7	32.9
4	21.8	27.1	33.8	38.7
5	23.6	29.9	37.2	42.3
6	25.2	31.6	39.9	45.9
7	27.1	33.6	42.1	48.0
8	28.7	35.4	44.0	50.4
9	30.5	38.3	46.9	54.0
10	32.4	40.9	50.0	58.0
11	33.9	43.5	52.7	61.2
12	35.6	45.3	55.0	63.4
14	36.9	46.6	56.8	66.3
16	38.4	49.1	59.9	68.9
18	39.9	50.8	62.1	71.4
20	41.3	52.4	64.4	73.5
22	42.1	53.8	66.1	74.5
24	43.0	54.2	67.1	75.6

Time	F1	F2	F3	F4
1	0.16	0.19	0.22	0.24
2	0.17	0.21	0.25	0.28
3	0.19	0.24	0.29	0.33
4	0.22	0.27	0.34	0.39
5	0.24	0.30	0.37	0.42
6	0.25	0.32	0.40	0.46
7	0.27	0.34	0.42	0.48
8	0.29	0.35	0.44	0.50
9	0.30	0.38	0.47	0.54
10	0.32	0.41	0.50	0.58
11	0.34	0.44	0.53	0.61
12	0.36	0.45	0.55	0.63
14	0.37	0.47	0.57	0.66
16	0.38	0.49	0.60	0.69
18	0.40	0.51	0.62	0.71
20	0.41	0.52	0.64	0.73
22	0.42	0.54	0.66	0.74
24	0.43	0.54	0.67	0.76

Time	F1	F2	F3	F4
1	0.54	0.57	0.60	0.62
2	0.55	0.60	0.63	0.65
3	0.58	0.62	0.66	0.69
4	0.60	0.65	0.70	0.73
5	0.62	0.67	0.72	0.75
6	0.63	0.68	0.74	0.77
7	0.65	0.70	0.75	0.78
8	0.66	0.71	0.76	0.80
9	0.67	0.73	0.78	0.81
10	0.69	0.74	0.79	0.83
11	0.70	0.76	0.81	0.85
12	0.71	0.77	0.82	0.86
14	0.72	0.78	0.83	0.87
16	0.73	0.79	0.84	0.88
18	0.74	0.80	0.85	0.89
20	0.74	0.81	0.86	0.90
22	0.75	0.81	0.87	0.91
24	0.75	0.82	0.88	0.91

Time	F1	F2	F3	F4
1	0.46	0.43	0.40	0.38
2	0.45	0.40	0.37	0.35
3	0.42	0.38	0.34	0.31
4	0.40	0.35	0.30	0.27
5	0.38	0.33	0.28	0.25
6	0.37	0.32	0.26	0.23
7	0.35	0.30	0.25	0.22
8	0.34	0.29	0.24	0.20
9	0.33	0.27	0.22	0.19
10	0.31	0.26	0.21	0.17
11	0.30	0.24	0.19	0.15
12	0.29	0.23	0.18	0.14
14	0.28	0.22	0.17	0.13
16	0.27	0.21	0.16	0.12
18	0.26	0.20	0.15	0.11
20	0.26	0.19	0.14	0.10
22	0.25	0.19	0.13	0.09
24	0.25	0.18	0.12	0.09

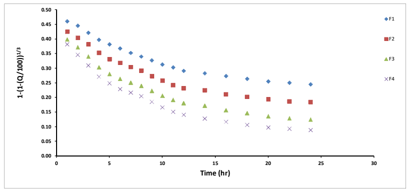

Time	R^2	R^2(part 1)	R^2(part 2)
F5	0.8939	0.9851	0.9863
F6	0.8891	0.9858	0.9706
F7	0.8766	0.9636	0.9799
F8	0.8540	0.9585	0.9629

Korsmeyer-Peppas mode

Time	F1	F2	F3	F4
1	15.7	19.0	21.8	23.6
2	17.0	21.1	24.8	27.9
3	19.4	23.6	28.7	32.9
4	21.8	27.1	33.8	38.7
5	23.6	29.9	37.2	42.3
6	25.2	31.6	39.9	45.9
7	27.1	33.6	42.1	48.0
8	28.7	35.4	44.0	50.4
9	30.5	38.3	46.9	54.0
10	32.4	40.9	50.0	58.0
11	33.9	43.5	52.7	61.2
12	35.6	45.3	55.0	63.4
14	36.9	46.6	56.8	66.3
16	38.4	49.1	59.9	68.9
18	39.9	50.8	62.1	71.4
20	41.3	52.4	64.4	73.5
22	42.1	53.8	66.1	74.5
24	43.0	54.2	67.1	75.6

Time	F1	F2	F3	F4
1	2.8	2.9	3.1	3.2
2	2.8	3.0	3.2	3.3
3	3.0	3.2	3.4	3.5
4	3.1	3.3	3.5	3.7
5	3.2	3.4	3.6	3.7
6	3.2	3.5	3.7	3.8
7	3.3	3.5	3.7	3.9
8	3.4	3.6	3.8	3.9
9	3.4	3.6	3.8	4.0
10	3.5	3.7	3.9	4.1
11	3.5	3.8	4.0	4.1
12	3.6	3.8	4.0	4.1
14	3.6	3.8	4.0	4.2
16	3.6	3.9	4.1	4.2
18	3.7	3.9	4.1	4.3
20	3.7	4.0	4.2	4.3
22	3.7	4.0	4.2	4.3
24	3.8	4.0	4.2	4.3

Time	F1	F2	F3	F4
0.0	3.1	3.1	3.0	3.0
0.7	3.4	3.3	3.2	3.2
1.1	3.6	3.5	3.3	3.3
1.4	3.7	3.7	3.5	3.4
1.6	3.8	3.8	3.6	3.5
1.8	3.9	3.9	3.7	3.6
1.9	4.0	3.9	3.7	3.7
2.1	4.0	4.0	3.8	3.7
2.2	4.1	4.1	3.9	3.8
2.3	4.2	4.2	3.9	3.9
2.4	4.2	4.2	4.0	3.9
2.5	4.3	4.3	4.1	4.0
2.6	4.4	4.3	4.1	4.0
2.8	4.5	4.4	4.2	4.1
2.9	4.5	4.5	4.3	4.2
3.0	4.6	4.6	4.4	4.3
3.1	4.6	4.6	4.4	4.3
3.2	4.6	4.6	4.4	4.3

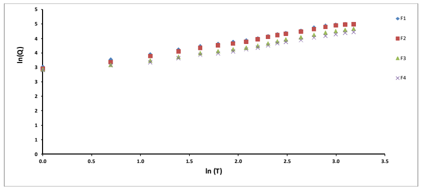

Time	Slope
F5	0.5018
F6	0.5223
F7	0.4793
F8	0.4514

Time	Slope
F5	0.4775
F6	0.5005
F7	0.4237
F8	0.4032

Weibull model

Time	F1	F2	F3	F4
1	15.7	19.0	21.8	23.6
2	17.0	21.1	24.8	27.9
3	19.4	23.6	28.7	32.9
4	21.8	27.1	33.8	38.7
5	23.6	29.9	37.2	42.3
6	25.2	31.6	39.9	45.9
7	27.1	33.6	42.1	48.0
8	28.7	35.4	44.0	50.4
9	30.5	38.3	46.9	54.0
10	32.4	40.9	50.0	58.0
11	33.9	43.5	52.7	61.2
12	35.6	45.3	55.0	63.4
14	36.9	46.6	56.8	66.3
16	38.4	49.1	59.9	68.9
18	39.9	50.8	62.1	71.4
20	41.3	52.4	64.4	73.5
22	42.1	53.8	66.1	74.5
24	43.0	54.2	67.1	75.6

Time	F1	F2	F3	F4
1	0.16	0.19	0.22	0.24
2	0.17	0.21	0.25	0.28
3	0.19	0.24	0.29	0.33
4	0.22	0.27	0.34	0.39
5	0.24	0.30	0.37	0.42
6	0.25	0.32	0.40	0.46
7	0.27	0.34	0.42	0.48
8	0.29	0.35	0.44	0.50
9	0.30	0.38	0.47	0.54
10	0.32	0.41	0.50	0.58
11	0.34	0.44	0.53	0.61
12	0.36	0.45	0.55	0.63
14	0.37	0.47	0.57	0.66
16	0.38	0.49	0.60	0.69
18	0.40	0.51	0.62	0.71
20	0.41	0.52	0.64	0.73
22	0.42	0.54	0.66	0.74
24	0.43	0.54	0.67	0.76

Time	F1	F2	F3	F4
1	0.17	0.21	0.25	0.27
2	0.19	0.24	0.28	0.33
3	0.22	0.27	0.34	0.40
4	0.25	0.32	0.41	0.49
5	0.27	0.35	0.46	0.55
6	0.29	0.38	0.51	0.61
7	0.32	0.41	0.55	0.65
8	0.34	0.44	0.58	0.70
9	0.36	0.48	0.63	0.78
10	0.39	0.53	0.69	0.87
11	0.41	0.57	0.75	0.95
12	0.44	0.60	0.80	1.00
14	0.46	0.63	0.84	1.09
16	0.48	0.68	0.91	1.17
18	0.51	0.71	0.97	1.25
20	0.53	0.74	1.03	1.33
22	0.55	0.77	1.08	1.37
24		0.78	1.11	1.41

Time	F1	F2	F3	F4
0.00	-1.77	-1.56	-1.41	-1.31
0.69	-1.68	-1.44	-1.26	-1.12
1.10	-1.54	-1.31	-1.08	-0.92
1.39	-1.40	-1.15	-0.89	-0.71
1.61	-1.31	-1.04	-0.77	-0.60
1.79	-1.24	-0.97	-0.68	-0.49
1.95	-1.15	-0.89	-0.60	-0.42
2.08	-1.08	-0.83	-0.55	-0.36
2.20	-1.01	-0.73	-0.46	-0.25
2.30	-0.94	-0.64	-0.37	-0.14
2.40	-0.88	-0.56	-0.29	-0.06
2.48	-0.82	-0.51	-0.22	0.00
2.64	-0.78	-0.47	-0.17	0.08
2.77	-0.73	-0.39	-0.09	0.16
2.89	-0.68	-0.34	-0.03	0.22
3.00	-0.63	-0.30	0.03	0.28
3.09	-0.60	-0.26	0.08	0.31
3.18		-0.25	0.11	0.34

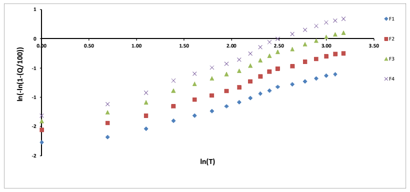

Time	R^2	R^2(part 1)	R^2(part 2)
F5	0.8980	0.9804	0.9929
F6	0.8872	0.9830	0.9719
F7	0.8865	0.9629	0.9833
F8	0.8779	0.9620	0.9711
