# Supplementary material for: Correction: Formulation and In Vitro, In Vivo Evaluation of Effervescent Floating Sustained-Release Imatinib Mesylate Tablet
Source: PLoS One. 2022 Sep 21;17(9):e0275144. doi: 10.1371/journal.pone.0275144 (PMC9491528; doi:10.1371/journal.pone.0275144)
Supplement: S7 File — (PDF) [file pone.0275144.s007.pdf]

Zero Order

Time	F5	F6	F7	F8	Column1	Column2	Column3	Column4
1	22.7	21.2	20.9	20.3				
2	29.2	26.8	24.3	23.9				
3	34.9	33.1	28.2	26.6				
4	41.2	38.6	32.1	30.7				
5	46.3	43.8	36.6	34.3				
6	50.0	47.5	39.1	36.3				
7	53.7	50.8	41.6	38.7				
8	56.6	54.2	44.0	41.8				
9	60.3	58.9	47.3	44.3				
10	65.2	64.0	51.0	47.8				
11	69.7	68.2	54.8	51.3				
12	73.2	71.4	58.3	53.6				
14	79.0	76.9	62.8	57.2				
16	88.1	83.8	68.7	62.4				
18	93.8	90.1	73.8	66.3				
20	97.4	95.3	78.0	70.2				
22	99.4	98.0	81.2	73.6				
24	100.0	99.7	84.7	76.5				

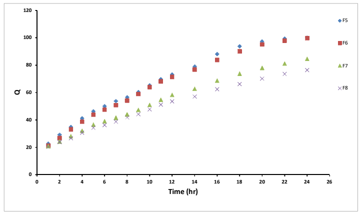

Time	R^2	R^2(part 1)	R^2(part 2)
F1			
F2			
F3			
F4			
F5	0.9668	0.9887	0.9155
F6	0.9733	0.9927	0.9652
F7	0.9887	0.9957	0.9891
F8	0.9870	0.9969	0.9937

	F5	F6	F7	F8
Zero Order	0.9668	0.9733	0.9887	0.9870
First Order	0.8517	0.8333	0.9867	0.9965
Higuchi model	0.9924	0.9948	0.9882	0.9911
Hixson-Crowell model	0.9120	0.9162	0.9508	0.9469
Weibull model	0.9878	0.9860	0.9709	0.9584

Part 1

	F5	F6	F7	F8	Column1	Column2	Column3	Column4
Zero Order	0.9887	0.9927	0.9957	0.9969				
First Order	0.9903	0.9894	0.9905	0.9944				
Higuchi model	0.9944	0.9913	0.9820	0.9827				
Hixson-Crowell model	0.9561	0.9636	0.9807	0.9835				
Weibull model	0.9647	0.9695	0.9801	0.9819				

Part II

	F5	F6	F7	F8
Zero Order	0.9155	0.9652	0.9891	0.9937
First Order	0.9333	0.8874	0.9969	0.9980
Higuchi model	0.9392	0.9790	0.9958	0.9949
Hixson-Crowell model	0.9044	0.9567	0.9818	0.9828
Weibull model	0.99350	0.98489	0.99446	0.98998

First Order

Time	F5	F6	F7	F8
1	77.3	78.8	79.1	79.7
2	70.8	73.2	75.7	76.1
3	65.1	66.9	71.8	73.4
4	58.8	61.4	67.9	69.3
5	53.7	56.2	63.4	65.7
6	50.0	52.5	60.9	63.7
7	46.3	49.2	58.4	61.3
8	43.4	45.8	56.0	58.2
9	39.7	41.1	52.7	55.8
10	34.8	36.0	49.0	52.2
11	30.3	31.8	45.2	48.7
12	26.8	28.6	41.7	46.4
14	21.0	23.1	37.2	42.8
16	11.9	16.2	31.3	37.6
18	6.2	9.9	26.2	33.7
20	2.6	4.7	22.0	29.8
22	0.6	2.0	18.8	26.4
24		0.3	15.4	23.6

Time	F5	F6	F7	F8
1	4.3	4.4	4.4	4.4
2	4.3	4.3	4.3	4.3
3	4.2	4.2	4.3	4.3
4	4.1	4.1	4.2	4.2
5	4.0	4.0	4.1	4.2
6	3.9	4.0	4.1	4.2
7	3.8	3.9	4.1	4.1
8	3.8	3.8	4.0	4.1
9	3.7	3.7	4.0	4.0
10	3.5	3.6	3.9	4.0
11	3.4	3.5	3.8	3.9
12	3.3	3.4	3.7	3.8
14	3.0	3.1	3.6	3.8
16	2.5	2.8	3.4	3.6
18	1.8	2.3	3.3	3.5
20	1.0	1.6	3.1	3.4
22	-0.5	0.7	2.9	3.3
24		-1.2	2.7	3.2

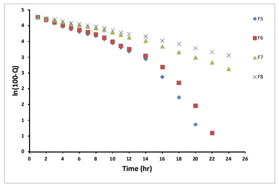

Time	R^2	R^2(part 1)	R^2(part 2)
F5	0.8517	0.9903	0.9333
F6	0.8333	0.9894	0.8874
F7	0.9867	0.9905	0.9969
F8	0.9965	0.9944	0.9980

Higuchi model

Time	F5	F6	F7	F8
1	22.7	21.2	20.9	20.3
2	29.2	26.8	24.3	23.9
3	34.9	33.1	28.2	26.6
4	41.2	38.6	32.1	30.7
5	46.3	43.8	36.6	34.3
6	50.0	47.5	39.1	36.3
7	53.7	50.8	41.6	38.7
8	56.6	54.2	44.0	41.8
9	60.3	58.9	47.3	44.3
10	65.2	64.0	51.0	47.8
11	69.7	68.2	54.8	51.3
12	73.2	71.4	58.3	53.6
14	79.0	76.9	62.8	57.2
16	88.1	83.8	68.7	62.4
18	93.8	90.1	73.8	66.3
20	97.4	95.3	78.0	70.2
22	99.4	98.0	81.2	73.6
24	100.0	99.7	84.7	76.5

(Time)$^{0.}$	F5	F6	F7	F8
1.0	22.7	21.2	20.9	20.3
1.4	29.2	26.8	24.3	23.9
1.7	34.9	33.1	28.2	26.6
2.0	41.2	38.6	32.1	30.7
2.2	46.3	43.8	36.6	34.3
2.4	50.0	47.5	39.1	36.3
2.6	53.7	50.8	41.6	38.7
2.8	56.6	54.2	44.0	41.8
3.0	60.3	58.9	47.3	44.3
3.2	65.2	64.0	51.0	47.8
3.3	69.7	68.2	54.8	51.3
3.5	73.2	71.4	58.3	53.6
3.7	79.0	76.9	62.8	57.2
4.0	88.1	83.8	68.7	62.4
4.2	93.8	90.1	73.8	66.3
4.5	97.4	95.3	78.0	70.2
4.7	99.4	98.0	81.2	73.6
4.9	100.0	99.7	84.7	75.6

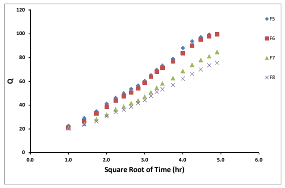

Time	R^2	R^2(part 1)	R^2(part 2)
F5	0.9924	0.9944	0.9392
F6	0.9948	0.9913	0.9790
F7	0.9882	0.9820	0.9958
F8	0.9911	0.9827	0.9949

Hixson-Crowell mode

Time	F5	F6	F7	F8
1	22.7	21.2	20.9	20.3
2	29.2	26.8	24.3	23.9
3	34.9	33.1	28.2	26.6
4	41.2	38.6	32.1	30.7
5	46.3	43.8	36.6	34.3
6	50.0	47.5	39.1	36.3
7	53.7	50.8	41.6	38.7
8	56.6	54.2	44.0	41.8
9	60.3	58.9	47.3	44.3
10	65.2	64.0	51.0	47.8
11	69.7	68.2	54.8	51.3
12	73.2	71.4	58.3	53.6
14	79.0	76.9	62.8	57.2
16	88.1	83.8	68.7	62.4
18	93.8	90.1	73.8	66.3
20	97.4	95.3	78.0	70.2
22	99.4	98.0	81.2	73.6
24	100.0	99.7	84.7	75.6

Time	F5	F6	F7	F8
1	0.23	0.21	0.21	0.20
2	0.29	0.27	0.24	0.24
3	0.35	0.33	0.28	0.27
4	0.41	0.39	0.32	0.31
5	0.46	0.44	0.37	0.34
6	0.50	0.48	0.39	0.36
7	0.54	0.51	0.42	0.39
8	0.57	0.54	0.44	0.42
9	0.60	0.59	0.47	0.44
10	0.65	0.64	0.51	0.48
11	0.70	0.68	0.55	0.51
12	0.73	0.71	0.58	0.54
14	0.79	0.77	0.63	0.57
16	0.88	0.84	0.69	0.62
18	0.94	0.90	0.74	0.66
20	0.97	0.95	0.78	0.70
22	0.99	0.98	0.81	0.74
24	1.00	1.00	0.85	0.76

Time	F5	F6	F7	F8
1	0.61	0.60	0.59	0.59
2	0.66	0.64	0.62	0.62
3	0.70	0.69	0.66	0.64
4	0.74	0.73	0.68	0.67
5	0.77	0.76	0.72	0.70
6	0.79	0.78	0.73	0.71
7	0.81	0.80	0.75	0.73
8	0.83	0.82	0.76	0.75
9	0.85	0.84	0.78	0.76
10	0.87	0.86	0.80	0.78
11	0.89	0.88	0.82	0.80
12	0.90	0.89	0.84	0.81
14	0.92	0.92	0.86	0.83
16	0.96	0.94	0.88	0.85
18	0.98	0.97	0.90	0.87
20	0.99	0.98	0.92	0.89
22	1.00	0.99	0.93	0.90
24	1.00	1.00	0.95	0.91

Time	F5	F6	F7	F8
1	0.39	0.40	0.41	0.41
2	0.34	0.36	0.38	0.38
3	0.30	0.31	0.34	0.36
4	0.26	0.27	0.32	0.33
5	0.23	0.24	0.28	0.30
6	0.21	0.22	0.27	0.29
7	0.19	0.20	0.25	0.27
8	0.17	0.18	0.24	0.25
9	0.15	0.16	0.22	0.24
10	0.13	0.14	0.20	0.22
11	0.11	0.12	0.18	0.20
12	0.10	0.11	0.16	0.19
14	0.08	0.08	0.14	0.17
16	0.04	0.06	0.12	0.15
18	0.02	0.03	0.10	0.13
20	0.01	0.02	0.08	0.11
22	0.00	0.01	0.07	0.10
24	0.00	0.00	0.05	0.09

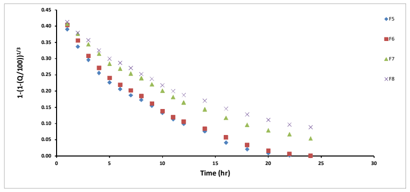

Time	R^2	R^2(part 1)	R^2(part 2)
F5	0.9120	0.9561	0.9044
F6	0.9162	0.9636	0.9567
F7	0.9508	0.9807	0.9818
F8	0.9469	0.9835	0.9828

Korsmeyer-Peppas mode

Time	F5	F6	F7	F8
1	22.7	21.2	20.9	20.3
2	29.2	26.8	24.3	23.9
3	34.9	33.1	28.2	26.6
4	41.2	38.6	32.1	30.7
5	46.3	43.8	36.6	34.3
6	50.0	47.5	39.1	36.3
7	53.7	50.8	41.6	38.7
8	56.6	54.2	44.0	41.8
9	60.3	58.9	47.3	44.3
10	65.2	64.0	51.0	47.8
11	69.7	68.2	54.8	51.3
12	73.2	71.4	58.3	53.6
14	79.0	76.9	62.8	57.2
16	88.1	83.8	68.7	62.4
18	93.8	90.1	73.8	66.3
20	97.4	95.3	78.0	70.2
22	99.4	98.0	81.2	73.6
24	100.0	99.7	84.7	75.6

Time	F5	F6	F7	F8
1	3.1	3.1	3.0	3.0
2	3.4	3.3	3.2	3.2
3	3.6	3.5	3.3	3.3
4	3.7	3.7	3.5	3.4
5	3.8	3.8	3.6	3.5
6	3.9	3.9	3.7	3.6
7	4.0	3.9	3.7	3.7
8	4.0	4.0	3.8	3.7
9	4.1	4.1	3.9	3.8
10	4.2	4.2	3.9	3.9
11	4.2	4.2	4.0	3.9
12	4.3	4.3	4.1	4.0
14	4.4	4.3	4.1	4.0
16	4.5	4.4	4.2	4.1
18	4.5	4.5	4.3	4.2
20	4.6	4.6	4.4	4.3
22	4.6	4.6	4.4	4.3
24	4.6	4.6	4.4	4.3

Time	F5	F6	F7	F8
0.0	3.1	3.1	3.0	3.0
0.7	3.4	3.3	3.2	3.2
1.1	3.6	3.5	3.3	3.3
1.4	3.7	3.7	3.5	3.4
1.6	3.8	3.8	3.6	3.5
1.8	3.9	3.9	3.7	3.6
1.9	4.0	3.9	3.7	3.7
2.1	4.0	4.0	3.8	3.7
2.2	4.1	4.1	3.9	3.8
2.3	4.2	4.2	3.9	3.9
2.4	4.2	4.2	4.0	3.9
2.5	4.3	4.3	4.1	4.0
2.6	4.4	4.3	4.1	4.0
2.8	4.5	4.4	4.2	4.1
2.9	4.5	4.5	4.3	4.2
3.0	4.6	4.6	4.4	4.3
3.1	4.6	4.6	4.4	4.3
3.2	4.6	4.6	4.4	4.3

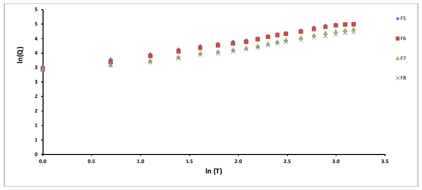

Time	Slope
F5	0.5018
F6	0.5223
F7	0.4793
F8	0.4514

Time	Slope
F5	0.4775
F6	0.5005
F7	0.4237
F8	0.4032

Weibull model

Time	F5	F6	F7	F8
1	22.7	21.2	20.9	20.3
2	29.2	26.8	24.3	23.9
3	34.9	33.1	28.2	26.6
4	41.2	38.6	32.1	30.7
5	46.3	43.8	36.6	34.3
6	50.0	47.5	39.1	36.3
7	53.7	50.8	41.6	38.7
8	56.6	54.2	44.0	41.8
9	60.3	58.9	47.3	44.3
10	65.2	64.0	51.0	47.8
11	69.7	68.2	54.8	51.3
12	73.2	71.4	58.3	53.6
14	79.0	76.9	62.8	57.2
16	88.1	83.8	68.7	62.4
18	93.8	90.1	73.8	66.3
20	97.4	95.3	78.0	70.2
22	99.4	98.0	81.2	73.6
24	100.0	99.7	84.7	75.6

Time	F5	F6	F7	F8
1	0.23	0.21	0.21	0.20
2	0.29	0.27	0.24	0.24
3	0.35	0.33	0.28	0.27
4	0.41	0.39	0.32	0.31
5	0.46	0.44	0.37	0.34
6	0.50	0.48	0.39	0.36
7	0.54	0.51	0.42	0.39
8	0.57	0.54	0.44	0.42
9	0.60	0.59	0.47	0.44
10	0.65	0.64	0.51	0.48
11	0.70	0.68	0.55	0.51
12	0.73	0.71	0.58	0.54
14	0.79	0.77	0.63	0.57
16	0.88	0.84	0.69	0.62
18	0.94	0.90	0.74	0.66
20	0.97	0.95	0.78	0.70
22	0.99	0.98	0.81	0.74
24	1.00	1.00	0.85	0.76

Time	F5	F6	F7	F8
1	0.26	0.24	0.23	0.23
2	0.34	0.31	0.28	0.27
3	0.43	0.40	0.33	0.31
4	0.53	0.49	0.39	0.37
5	0.62	0.58	0.46	0.42
6	0.69	0.64	0.50	0.45
7	0.77	0.71	0.54	0.49
8	0.83	0.78	0.58	0.54
9	0.92	0.89	0.64	0.58
10	1.06	1.02	0.71	0.65
11	1.19	1.15	0.79	0.72
12	1.32	1.25	0.88	0.77
14	1.56	1.46	0.99	0.85
16	2.13	1.82	1.16	0.98
18	2.78	2.32	1.34	1.09
20	3.64	3.05	1.52	1.21
22	5.13	3.91	1.67	1.33
24		5.84	1.87	1.41

Time	F5	F6	F7	F8
0.00	-1.36	-1.43	-1.45	-1.49
0.69	-1.06	-1.17	-1.28	-1.30
1.10	-0.85	-0.91	-1.10	-1.17
1.39	-0.63	-0.72	-0.95	-1.00
1.61	-0.48	-0.55	-0.78	-0.87
1.79	-0.37	-0.44	-0.70	-0.80
1.95	-0.26	-0.34	-0.62	-0.71
2.08	-0.18	-0.25	-0.55	-0.61
2.20	-0.08	-0.12	-0.45	-0.54
2.30	0.06	0.02	-0.34	-0.43
2.40	0.18	0.14	-0.23	-0.33
2.48	0.28	0.23	-0.13	-0.27
2.64	0.45	0.38	-0.01	-0.17
2.77	0.76	0.60	0.15	-0.02
2.89	1.02	0.84	0.29	0.08
3.00	1.29	1.11	0.42	0.19
3.09	1.64	1.36	0.51	0.29
3.18		1.77	0.63	0.34

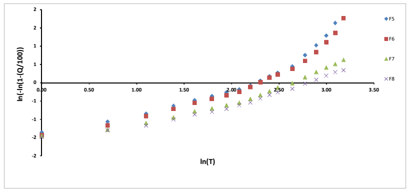

Time	R^2	R^2(part 1)	R^2(part 2)
F5	0.9878	0.9647	0.9935
F6	0.9860	0.9695	0.9849
F7	0.9709	0.9801	0.9945
F8	0.9584	0.9819	0.9900
