# Supplementary material for: Correction: Formulation and In Vitro, In Vivo Evaluation of Effervescent Floating Sustained-Release Imatinib Mesylate Tablet
Source: PLoS One. 2022 Sep 21;17(9):e0275144. doi: 10.1371/journal.pone.0275144 (PMC9491528; doi:10.1371/journal.pone.0275144)
Supplement: S3 File — (PDF) [file pone.0275144.s003.pdf]

imatinib+CGP		GLEEVIC			Ugr/ml								
	0.5	1	2	3	4	6	8	10	12	18	24	36	48	72
1	21.14	51.70	68.83	79.33	69.62	58.91	43.68	42.07	41.83	30.12	19.82	10.67	6.65	2.45
2	17.02	40.81	61.65	75.83	59.57	53.53	50.04	47.91	35.04	31.74	21.70	14.78	7.90	1.04
3	27.40	49.94	71.88	68.52	64.97	51.02	43.87	38.40	35.98	30.52	18.38	13.30	6.15	0.72
4	22.26	48.59	76.61	73.01	71.15	57.24	49.22	39.62	42.34	25.38	23.43	11.58	9.62	3.02
Mean	22.0	47.8	69.7	74.2	66.3	55.2	46.7	42.0	38.8	29.4	20.8	12.6	7.6	1.8
SD	4.3	4.8	6.3	4.6	5.2	3.6	3.4	4.2	3.8	2.8	2.2	1.8	1.5	1.1
SEM	2.1	2.4	3.1	2.3	2.6	1.8	1.7	2.1	1.9	1.4	1.1	0.9	0.8	0.6
CV	19.5	10.1	9.0	6.2	7.9	6.5	7.3	10.1	9.8	9.5	10.6	14.5	20.4	61.1
Min	17.0	40.8	61.7	68.5	59.6	51.0	43.7	38.4	35.0	25.4	18.4	10.7	6.2	0.7
Max	27.4	51.7	76.6	79.3	71.2	58.9	50.0	47.9	42.3	31.7	23.4	14.8	9.6	3.0

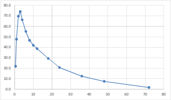

	AUC(0-0.5)	AUC(0.5-1)	AUC(1-2)	AUC(2-3)	AUC(3-4)	AUC(4-6)	AUC(6-8)	AUC(8-10)	AUC(10-12	AUC(12-18	AUC(18-24	AUC(24-36	AUC(36-48	AUC(48-72	AUC(0-t)
A	5.3	18.2	60.3	74.1	74.5	128.5	102.6	85.7	83.9	215.9	149.8	182.9	103.9	109.2	1394.8
B	4.3	14.5	51.2	275.0	135.4	113.1	12.5	98.0	83.0	200.3	160.3	218.9	136.1	107.3	1609.7
C	6.9	19.3	60.9	280.8	133.5	232.0	11.0	20.6	37.2	133.0	48.9	63.4	116.7	41.2	744.3
D	5.6	17.7	62.6	299.2	144.2	256.8	12.3	22.2	41.0	135.4	48.8	70.0	127.2	75.8	798.4

	LN4	LN 6	LN 8	LN 10	LN 12	LN 18	LN 24	LN 36	LN 48	LN 72	Slope	Ke	T½	Clast/ke	AUC(0-∞)	Cmax	AUMC(0-t)	Clast*t/ke	Clast/ke^2	'ke^2)+(Clas	AUMC(0-∞)
A	4.2	4.1	3.8	3.7	3.7	3.4	3.0	2.4	1.9	0.9	-0.1	0.1	13.2	46.6	1441.4	79.3	!!	3358.2	887.9	4246.1	#VALUE!
B	4.3	4.1	4.0	3.9	3.9	3.6	3.5	3.1	2.7	2.1	-0.1	0.1	6.5	499.6	2109.4	75.8	!!	5995.7	0.1	5995.8	#VALUE!
C	4.2	4.2	3.9	3.8	3.6	3.6	3.4	2.9	2.6	1.8	-0.1	0.1	6.8	502.5	1246.9	71.9	2863.8	6030.5	0.1	6030.6	8894.3
D	4.3	4.3	4.0	3.9	3.7	3.7	3.2	3.2	2.4	2.3	-0.1	0.1	8.1	672.9	1471.3	76.6	2214.3	8074.8	0.1	8074.8	10289.2

AUC (0-24)
998.7

	Cmax	Tmax	Ke	T½	AUC(0-t)	AUC(0-∞)	MRT
A	79.3	2.0	0.1	13.2	1394.8	786.5	#VALUE!
B	75.8	2.0	0.1	6.5	1609.7	352.2	#VALUE!
C	71.9	2.0	0.1	6.8	744.3	784.1	7.1
D	76.6	1.0	0.1	8.1	798.4	774.6	7.0
Mean	75.9	1.8	0.1	8.7	1136.8	674.3	#VALUE!
SD	3.1	0.5	0.0	3.1	431.6	214.8	#VALUE!
SEM	1.5	0.3	0.0	1.6	215.8	107.4	#VALUE!
CV	4.1	28.6	28.4	35.9	38.0	31.9	#VALUE!
Min	71.9	1.0	0.1	6.5	744.3	352.2	#VALUE!
Max	79.3	2.0	0.1	13.2	1609.7	786.5	#VALUE!

Cmax (µ	tmax (h)	t1/2 (h)	AUC (0-2	AUC (0-t	AUC(0-∞)
74.23	2.5-3.5	14.5	970.9527	1406.393	1444.547

		FSRT												Ugr/ml
	0.5	1	2	3	4	6	8	10	12	18	24	36	48	72
1	5.05	18.37	33.57	39.38	47.45	52.06	60.38	58.85	59.25	62.82	53.92	25.33	8.23	0.63
2	10.4	23.11	30.58	44.57	50.99	62.74	61.52	65.34	60.97	60.7	51.39	26.61	15.84	0.26
3	8.44	18.56	32.63	42.79	48.16	55.36	53.27	58.41	65.17	56.33	52.74	30.19	9.7	0
4	9.62	20.19	26.21	46.1	55.52	56.98	62.73	62.69	60.8	58.78	48.5	27.42	11.12	1.4
Mean	8.4	20.1	30.7	43.2	50.5	56.8	59.5	61.3	61.5	59.7	51.6	27.4	11.2	0.6
SD	2.4	2.2	3.3	2.9	3.7	4.5	4.2	3.3	2.5	2.8	2.3	2.1	3.3	0.6
SEM	1.2	1.1	1.6	1.4	1.8	2.2	2.1	1.6	1.3	1.4	1.2	1.0	1.6	0.3
CV	28.2	10.9	10.6	6.7	7.2	7.9	7.1	5.4	4.1	4.6	4.5	7.5	29.4	106.4
Min	5.1	18.4	26.2	39.4	47.5	52.1	53.3	58.4	59.3	56.3	48.5	25.3	8.2	0.0
Max	10.4	23.1	33.6	46.1	55.5	62.7	62.7	65.3	65.2	62.8	53.9	30.2	15.8	1.4

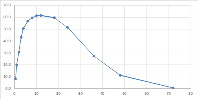

	AUC(0-0.5)	AUC(0.5-1)	AUC(1-2)	AUC(2-3)	AUC(3-4)	AUC(4-6)	AUC(6-8)	AUC(8-10)	AUC(10-12	AUC(12-18	AUC(18-24	AUC(24-36	AUC(36-48	AUC(48-72	AUC(0-t)
A	1.3	5.9	26.0	36.5	43.4	99.5	112.4	119.2	118.1	366.2	350.2	475.5	201.4	106.3	2061.9
B	2.6	8.4	26.8	150.3	95.6	113.7	15.4	126.9	126.3	365.0	336.3	468.0	254.7	193.2	2283.1
C	2.1	6.8	25.6	150.8	91.0	207.0	13.3	27.9	61.8	243.0	109.1	165.9	239.3	58.2	496.6
D	2.4	7.5	23.2	144.6	101.6	225.0	15.7	31.4	61.7	239.2	107.3	151.8	231.2	75.1	520.0

	LN 10	LN 12	LN 18	LN 24	LN 36	LN 48	LN 72	Slope	Ke	T½	Clast/ke	AUC(0-∞)	Cmax	AUMC(0-t)	Clast*t/ke	Clast/ke^2	'ke^2)+(Clas	AUMC(0-∞)
A	4.1	4.1	4.1	4.0	3.2	2.1	-0.5	-0.1	0.1	13.5	12.3	2074.2	62.8	!!	886.9	240.8	1127.7	#VALUE!
B	4.1	4.2	4.1	4.1	3.9	3.3	2.8	#REF!	#REF!	#REF!	#REF!	#REF!	62.7	!!	#REF!	0.1	#REF!	#REF!
C	4.0	4.1	4.2	4.0	4.0	3.4	2.3	#REF!	#REF!	#REF!	#REF!	#REF!	55.4	2863.8	#REF!	0.1	#REF!	#REF!
D	4.1	4.1	4.1	4.1	3.9	3.3	2.4	#REF!	#REF!	#REF!	#REF!	#REF!	62.7	2214.3	#REF!	0.1	#REF!	#REF!

AUC (0-24)
1278.7

	Cmax	Tmax	Ke	T½	AUC(0-t)	AUC(0-∞)	MRT
A	98.3	8.0	AUC(36-48	AUC(48-72	AUC(8-10)	#REF!	#VALUE!
B	#REF!	6.0	201.36	106.3	119.2	#REF!	#REF!
C	#REF!	6.0	254.70	193.2	126.9	#REF!	#REF!
D	#REF!	6.0	239.34	58.2	27.9	#REF!	#REF!
Mean	#REF!	6.5	231.80	119.2	91.3	#REF!	#VALUE!
SD	#REF!	1.0	27.46	68.4	55.1	#REF!	#VALUE!
SEM	#REF!	0.5	13.73	34.2	27.5	#REF!	#VALUE!
CV	#REF!	15.4	11.8	57.4	60.3	#REF!	#VALUE!
Min	#REF!	6.0	201.4	58.2	27.9	#REF!	#VALUE!
Max	#REF!	8.0	254.7	193.2	126.9	#REF!	#VALUE!

Cmax (µ	tmax (h)	t1/2 (h)	AUC (0-2	AUC (0-t	AUC(0-∞)
61.52	10-11.5	34.3	1283.233	2131.293	2145.493
